# Comparative effectiveness of interventions to facilitate deprescription of benzodiazepines and other sedative hypnotics: systematic review and meta-analysis

**DOI:** 10.1136/bmj-2024-081336

**Published:** 2025-06-17

**Authors:** Dena Zeraatkar, Sumanth Kumbargere Nagraj, Michael Ling, Tanvir Jassal, Sarah Kirsh, João Pedro Lima, Tyler Pitre, Rachel Couban, Muizz Hussain, Siri Seterelv, Stijn Van de Velde, Katarzyna Gustavsson, Adam Wichniak, Carole E Aubert, Antoine Christiaens, Anne Spinewine, Thomas Agoritsas

**Affiliations:** 1 Department of Anesthesia, McMaster University, Hamilton, ON, Canada; 2 Department Health Research Methods, Evidence, and Impact, McMaster University, Hamilton, ON, Canada; 3 MAGIC Evidence Ecosystem Foundation, Oslo, Norway; 4 Division of Respirology; Department of Medicine, University of Toronto, Toronto, ON, Canada; 5 Department of Medicine, McMaster University, Hamilton, ON, Canada; 6 Institute of Psychiatry and Neurology, Third Department of Psychiatry and Sleep Medicine Center, Warsaw, Poland; 7 Department of Science and Evaluation, Medical Research Agency, Warsaw, Poland; 8 Department of General Internal Medicine, Inselspital, Bern University Hospital, University of Bern, Bern, Switzerland; 9 Institute of Primary Health Care (BIHAM), University of Bern, Bern, Switzerland; 10 Fund for Scientific Research – FNRS, Belgium; 11 Clinical Pharmacy and Pharmacoepidemiology Research Group, Louvain Drug Research Institute, Louvain, Belgium; 12 CHU UCL Namur, Pharmacy department, Yvoir, Belgium; 13 Division General Internal Medicine, Department of Medicine, University Hospitals of Geneva, Geneva, Switzerland

## Abstract

**Objective:**

To review evidence from randomised trials assessing the effectiveness of strategies to deprescribe benzodiazepines and closely related sedative hypnotics (BSH).

**Design:**

Systematic review and meta-analysis of randomised controlled trials.

**Data sources:**

MEDLINE, Embase, CINAHL, PsycInfo, and CENTRAL, searched from inception to August 2024, and reference lists of included studies and similar systematic reviews.

**Eligibility criteria for selecting studies:**

Eligible studies randomised adults using BSH for insomnia to interventions aimed at deprescribing BSH, strategies to implement these interventions in healthcare settings, or usual care or placebo.

**Methods:**

Reviewers worked independently and in duplicate to screen search results, extract data, and assess risk of bias. Similar interventions were grouped together, frequentist random effects meta-analysis was conducted, and the certainty of evidence was assessed using the GRADE (Grading of Recommendations Assessment, Development, and Evaluation) approach.

**Results:**

The review identified 58 publications reporting on 49 unique trials with more than 39 000 patients. Interventions were classified into the following categories: tapering, patient education, physician education, combined patient and physician education, cognitive behavioural therapy, medication review, mindfulness, motivational interviewing, pharmacist led interventions, and drug assisted tapering and withdrawal. Low certainty evidence suggests that education of patients (144 (95% confidence interval 61 to 246) more per 1000 patients), medication review (104 (34 to 191) more), and a pharmacist led educational intervention (491 (234 to 928) more) may increase the proportion of patients who discontinue BSH compared with usual care. Moderate certainty evidence suggests that education of patients probably has little or no effect on physical function, mental health, and signs and symptoms of insomnia. No evidence was found regarding these other outcomes for medication review or for the pharmacist led educational intervention. No compelling evidence was found that other interventions may help patients to discontinue BSH. Moreover, no high or moderate certainty evidence was found that any of the interventions caused an increase in dropouts. Finally, low certainty evidence suggests that multicomponent interventions may be more effective at facilitating discontinuation of BSH than single component interventions.

**Conclusion:**

The evidence on the effectiveness of interventions to discontinue BSH is of low certainty. Educating patients, doing medication reviews, and a pharmacist led educational intervention may increase the proportion of patients who discontinue BSH.

## Introduction

Benzodiazepines and closely related sedative hypnotics (BSH) are commonly used to treat insomnia. Because these drugs may cause falls, cognitive impairment, and dependence, particularly in older and/or vulnerable people, guidelines advise healthcare providers to discontinue BSH.^1^ ^2^ ^3^ ^4^ ^5^ ^6^ ^7^ ^8^ ^9^ ^10^ ^11^ ^12^ ^13^ ^14^ ^15^ ^16^ ^17^ ^18^ ^19^ ^20^ ^21^ Nevertheless, prolonged use of BSH remains common, with associated harms, healthcare burdens, and costs.^22^ ^23^ ^24^ ^25^ ^26^

Research has documented diverse reasons for prolonged use of BSH. Patients report that they are not aware of the hazards of BSH or alternative drug and non-drug options to manage insomnia.^27^ ^28^ ^29^ ^30^ When patients attempt to discontinue BSH, they may be deterred by symptoms of physiological and psychological dependence, anxiety, or poor sleep.^30^ ^31^ ^32^ Healthcare providers report limited knowledge of alternatives to BSH, a lack of clarity on the harms of BSH, and insufficient evidence on optimal strategies to support the deprescription of BSH, defined as the planned, supervised, and safe process of dose reduction or complete cessation.^29^ ^33^

In response to these problems, this systematic review and meta-analysis identifies, pools, and appraises evidence from randomised trials assessing the effectiveness of interventions to facilitate deprescription of BSH and strategies for the implementation of such interventions in healthcare settings.

This review is part of the BMJ Rapid Recommendations project—a collaborative effort from the MAGIC Evidence Ecosystem Foundation (www.magicevidence.org) and *The BMJ* to produce trustworthy recommendations in response to practice changing evidence. This BMJ Rapid Recommendations project was triggered by BE-SAFE (BEnzodiazepine and sedative hypnotic use to improve patient SAFEty and quality of care)—a project funded by the European Union’s Horizon Europe research and innovation programme and the Swiss State Secretariat for Education, Research and Innovation to improve patient safety and prescription practices related to BSH for insomnia.^34^ The parallel BMJ Rapid Recommendations guideline panel, comprised of patient partners, methodologists, general practitioners, internists, geriatricians, a psychiatrist, a public health physician, a clinical pharmacist, and a social worker, helped to define the research question and the scope of the review. The panel included only people free of financial conflicts of interest, and potential intellectual conflicts were balanced among panel members.

## Methods

We developed a protocol for this systematic review and meta-analysis, which was reviewed and approved by the BE SAFE collaborators in February 2023 and registered online on Open Science Framework (https://osf.io/8em4p/files/osfstorage). We report our study according to the Preferred Reporting Items for Systematic Reviews and Meta-Analyses (PRISMA) checklist.^35^

### Eligibility criteria

Eligible studies randomised adult patients using BSH for insomnia to interventions aimed at deprescribing or discontinuation of BSH, strategies to implement these interventions, or standard care or placebo. Examples of eligible interventions include, but are not limited to, supervised tapering with or without drug support, education of patients about the harms of BSH, education of healthcare providers, behavioural interventions such as cognitive behavioural therapy, and system level interventions such as implementing new protocols or services in healthcare facilities. BSH drugs include but are not limited to benzodiazepines (for example, diazepam, alprazolam) and non-benzodiazepine “z-drugs” (for example, zolpidem, zaleplon). We did not restrict eligibility on the basis of duration of BSH use.

We anticipated that some trials would recruit patients who used BSH for a mix of different conditions and that some trials may not report the primary reason for which patients were using BSH. We included such trials unless explicit evidence showed that 60% or more of patients were using BSH for conditions other than insomnia. However, we did subgroup analyses investigating differences in results between trials in which patients were explicitly using BSH for insomnia and other trials in which the reason for using BSH was not reported.

Eligible designs included parallel, cluster, or crossover-by-cluster randomised trials. We excluded crossover trials because successful discontinuation of BSH in the first phase of the trial would render patients ineligible for subsequent phases of crossover. We did not restrict eligibility on the basis of language or time of publication. We excluded observational studies; systematic, scoping, or narrative reviews; trials that investigated interventions that only targeted discontinuation of non-BSH drugs; and trials that investigated the effectiveness of societal interventions (for example, messaging in the media).

We also excluded trials with fewer than 20 patients per arm. We anticipated that smaller trials would contribute little to meta-analyses, be at higher risk of publication bias, and more often include unrepresentative samples and trial arms that were prognostically imbalanced.^36^

### Search strategy

We worked with an experienced research librarian to devise and implement a search of MEDLINE, Embase, CINAHL, PsycInfo, and CENTRAL, from inception to August 2024 (supplement 1). Our search combined terms related to BSH, discontinuation and deprescription, and randomised trials. We supplemented our search by soliciting the parallel BMJ Rapid Recommendations guideline panel for additional eligible trials, doing forwards and backwards searching, and reviewing references of similar systematic reviews.^37^ ^38^ ^39^ ^40^ ^41^ ^42^ ^43^ ^44^ ^45^

### Screening

Following training and calibration exercises to ensure sufficient agreement, pairs of reviewers, working independently and in duplicate, screened the titles and abstracts of search records and subsequently the full texts of records deemed eligible at the title and abstract screening stage by using Covidence (https://www.covidence.org) online systematic review software. Reviewers resolved discrepancies by discussion or, when necessary, by adjudication with a third reviewer.

### Data extraction

Following training and calibration to ensure sufficient agreement, pairs of reviewers, working independently and in duplicate, extracted data from eligible trials into a structured Excel spreadsheet. We extracted data on characteristics of trials (for example, trial design, country, setting), patients (for example, age, sex, duration of BSH use), and interventions (for example, type, duration, intensity) and outcomes of interest.

A formal outcome prioritisation exercise with the parallel BMJ Rapid Recommendations panel directed our selection of outcomes.^46^ ^47^ Outcomes of interest included the proportion of patients who discontinue BSH, quality of life, physical function, mental health, cognitive function, signs and symptoms of insomnia, sleep efficiency, total sleep time, sleep onset latency, number of BSH prescriptions, drug-free nights, and dropouts of patients during the intervention.^48^ We expected that trials would measure and report these outcomes by using several different instruments. We extracted data for all validated instruments measuring our outcomes of interest. When trials reported both self-reported and laboratory tested discontinuation of BSH, we prioritised laboratory test results for their objectivity.

We anticipated that the effects of interventions might vary according to the age of patients and the duration of BSH use. We intended to extract data stratified by these factors, when reported, but did not identify any cases in which trials reported results stratified according to these factors.

We preferentially extracted intention-to-treat data without any imputations at the longest reported time of follow-up at which randomised groups were still preserved. For dichotomous outcomes, we extracted numbers of participants and events in each arm and relative effects (for example, relative risks) and associated confidence intervals. For continuous outcomes, we extracted number of participants, means, and measures of variability in each arm. We preferentially extracted the mean change in continuous outcomes when reported or, when not reported, the mean measure at follow-up.^49^ To facilitate meta-analyses, when trials reported medians or interquartile ranges for continuous outcomes, we converted these measures to means and standard deviations by using previously described methods.^50^

For cluster randomised trials, we additionally extracted the intracluster correlation coefficient—a measure of the degree to which patients within the same cluster (for example, patients in the same nursing home) are similar to each other in their response to an intervention compared with individuals from other clusters.^51^ For interventions targeting the discontinuation of several different classes of inappropriate medications, we extracted outcome data only for patients using BSH. When trials only reported data in figures, we used WebPlotDigitizer to extract data (https://automeris.io/WebPlotDigitizer/).

For publications in languages other than English, we planned to use Google Translate (https://translate.google.com/) to perform extractions and ask a native language speaker to confirm the extractions. We did not, however, find any publications in any language other than English. Reviewers resolved disagreements by discussion, or, when necessary, by adjudication with a third reviewer.

### Assessment of risk of bias

Following training and calibration, reviewers worked independently and in duplicate to assess risk of bias by using a modified version of the Cochrane endorsed Risk of Bias 2.0 tool (RoB 2.0) —the gold standard tool for assessing limitations in trials that may bias results.^52^ This tool assesses the risk of bias of trials across five domains: bias due to randomisation (for example, random sequence generation, allocation concealment), bias due to deviations from the intended intervention (for example, lack of blinding leading to imbalances in co-interventions across trial arms), bias due to missing outcome data (for example, attrition), bias due to measurement of the outcome (for example, unblinded outcome assessment), and selective reporting (for example, selective reporting of outcome measures on the basis of results). We additionally considered bias due to the timing of identification or recruitment of participants for cluster randomised trials and bias due to period and carryover effects for crossover-by-cluster trials.

To assess the risk of bias due to deviations from the intended intervention, we considered the effect of assignment rather than adherence to the intervention, as this effect is likely to be the observed effect in clinical settings and of the greatest interest to evidence users. We considered trials without masking of patients, healthcare providers, and investigators to be at high risk of bias for deviations from the intended intervention owing to the potential for differential care across trial arms. For example, we hypothesised that knowledge among healthcare providers that they are part of a randomised trial concerning the discontinuation of BSH could lead them to advise patients to stop using BSH regardless of the intervention to which participants have been randomised. Likewise, such information could make patients more likely to attempt to discontinue BSH independent of the intervention to which they have been randomised.

Our modified version of the RoB 2.0 tool includes the same domains, but with revised response options (that is, definitely low risk of bias, probably low risk of bias, probably high risk of bias, and definitely high risk of bias) and, in response to evidence suggesting that the original RoB 2.0 tool has poor inter-rater reliability,^53^ detailed guidance tailored to factors relevant for this review (for example, removing guidance for assessing risk of bias of adhering to the intervention, listing important co-interventions to consider). Reviewers resolved disagreements by discussion or by consultation with a third reviewer, if necessary.

### Data synthesis and analysis

A group of content experts and methodologists, with feedback from the wider guideline panel, classified similar interventions together for synthesis, considering the objective of the intervention, the target stakeholder (for example, patients, physicians, nurses, pharmacists), class of therapy (for example, cognitive behavioural therapy, mindfulness based therapy), and whether the intervention involved gradual or abrupt reduction in BSH dose, educational materials, or meetings with professionals. For analyses investigating the effects of cognitive behavioural therapy against usual care, we included trials that compared cognitive behavioural therapy against no additional interventions, cognitive behavioural therapy against tapering, the combination of cognitive behavioural therapy and tapering against tapering alone, and the combination of cognitive behavioural therapy and tapering against no additional interventions. We anticipated that all cognitive behavioural therapy interventions would include some suggestion of tapering. However, we did subgroup analyses to investigate differences in the effects of cognitive behavioural therapy when administered alone or with tapering and when compared against tapering or usual care.

We expected that interventions comprising more than one component would be more effective than interventions with a single component. Thus, we also classified interventions as multicomponent or single component to facilitate comparisons between the two. Multicomponent interventions included the combination of audit and feedback with education of physicians, cognitive behavioural therapy and tapering, education of both patients and physicians, and complex interventions that involved the introduction of a new professional role or new healthcare professionals in the healthcare setting or the training of healthcare professionals to perform additional tasks outside of their main scope of practice. Conversely, we classified cognitive behavioural therapy as a single component intervention as it comprises a distinct class of therapy.^54^ We classified the combination of tapering and education of patients and cognitive behavioural therapy alone or in combination with education of patients as single component interventions because tapering and cognitive behavioural therapy will ultimately always include an educational component. We also considered drug assisted tapering as a single component intervention.

We restricted comparisons of multicomponent and single component interventions to our most important outcome representing the effectiveness of interventions (the proportion of patients who discontinue BSH) and another outcome representing acceptability and safety of the intervention (the proportion of patients who discontinued the intervention).

When possible, for each unique comparison of interventions and outcome, we did frequentist random effects meta-analysis using the restricted maximum likelihood (REML) estimator—a conservative approach to meta-analysis—using data from the last point of follow-up from each trial at which randomised groups were still preserved.^55^ For comparisons for which meta-analysis was not possible, we planned to describe the range and distribution of results narratively, according to synthesis without meta-analysis (SWiM) guidelines.^56^ We did not, however, encounter comparisons for which we could not do quantitative synthesis.

We found that trials measured and reported results for physical function, mental health, and quality of life by using several different instruments. We pooled the results of each unique measure separately. We avoided using standardised mean differences owing to their potential to be influenced by differences in variability across trial populations.^57^ We also avoided converting units of one measure to another owing to potential differences in the range of the construct covered by each measure.^57^

For cluster trials that appropriately accounted for correlations within clusters, we used the reported results in the analysis. Otherwise, we adjusted trial results by the design effect, which we calculated using either the intraclass correlation coefficient reported in the trial or, if one was not reported, the median intraclass correlation coefficient across all included trials.^49^

For trials with multiple arms assessing the same intervention, we combined the number of participants and events for dichotomous outcomes and did fixed effect meta-analysis for the arms that assessed the same intervention for continuous outcomes. For trials with multiple arms assessing the same intervention that reported only relative effects or mean differences against a common comparator group, we pooled the arms assessing the same intervention together, using previously described methods to avoid double counting the comparator group.^58^

We summarised heterogeneity by using the I^2^ statistic and interpreted an I^2^ value of 0% to 40% as not important, 30% to 60% as moderate heterogeneity, 50% to 90% as substantial heterogeneity, and 75% to 100% as considerable heterogeneity.^49^ The I^2^ value is prone to misinterpretation as even small degrees of clinically unimportant inconsistency may translate to high I^2^ values if estimates from studies are precise.^59^ ^60^ Hence, we also considered the absolute magnitude of differences in estimates across studies. For analyses with 10 or more studies, we had planned to test for publication bias by visually inspecting funnel plots and Eggers tests, but all analyses included fewer than 10 studies.^61^

Like conventional meta-analysis, which pools results from studies assessing the same or similar research questions, network meta-analysis also pools study results but allows comparisons among three or more interventions simultaneously. We opted against network meta-analyses owing to the sparsity of evidence across outcomes and outcome measures and to concerns with transitivity or joint randomisability—a critical assumption of network meta-analysis.^62^ In particular, the types of interventions studied in trials varied across settings. For example, studies conducted in the community often focused on less intensive interventions such as patient education and tapering, whereas trials conducted in specialised settings studied more intensive interventions, such as introducing new healthcare professionals or training healthcare professionals to perform additional tasks outside of their main scope of practice. Given the likely differences in patient populations across these settings, comparisons between these interventions violate the assumption of transitivity requisite for network meta-analysis.

We used the meta and metafor packages in R (version 4.1.2) for all analyses and uploaded our data, code, and forest plots on Open Science Framework (https://osf.io/8em4p/files/osfstorage).^63^ ^64^

#### Subgroup analyses

We anticipated that the effects of interventions may vary according to several a priori specified factors—risk of bias, care setting (inpatients versus outpatients versus nursing homes), age (<65 versus ≥65), country (high income versus low/middle income), duration of BSH use (<median or ≥median across trials), and method for ascertaining BSH discontinuation (for example, patient report, medical records, or toxicology). To test for differences in effects according to these factors, we planned to do subgroup analyses. We were unable to do subgroup analyses based on care setting because of insufficient variation in care settings across trials reporting on similar interventions, age because trials did not report results stratified by age and large overlap existed in the age of participants across trials, country because almost all trials were performed in high income countries, and duration of BSH use because all trials did not report results stratified by duration of BSH use and large overlap existed in the duration of BSH use across trials.

We also hypothesised that the effects of interventions may vary according to more specific characteristics of the intervention (for example, pace at which patients were instructed to taper BSH, mode of delivery of cognitive behavioural therapy). In such cases, we did post hoc subgroup analyses to look for differences based on these factors. When we encountered situations in which a single study could contribute to more than one subgroup, we split the shared control group between the two subgroups to avoid double counting patients.^58^ We evaluated the credibility of subgroup effects by using the ICEMAN tool.^65^ In situations in which we had credible evidence of a subgroup effect, we planned to present stratified results based on the subgroup. We did not, however, identify any credible evidence of subgroup effects.

### Certainty of evidence and reporting

We assessed the certainty of evidence by using the GRADE (Grading of Recommendations Assessment, Development, and Evaluation) approach.^66^ For each comparison and outcome, we rated the certainty of evidence as either high, moderate, low, or very low on the basis of considerations of risk of bias (that is, study limitations), inconsistency (that is, heterogeneity in results across trials), indirectness (that is, differences between the questions investigated in studies and the question of interest), imprecision (that is, random error), and publication bias (that is, the tendency for studies with statistically significant results or positive results to be published, published faster, or published in journals with higher visibility).

High certainty evidence suggests that the estimated effect (the results from a rigorous systematic review and meta-analysis) is likely to be close to the true effect. Conversely, low or very low certainty evidence suggests that the estimated effect may in fact be substantially different from the true effect.

To make judgments about indirectness, we considered the context in which the trial was conducted. For non-drug interventions, we anticipated that evidence primarily comprised of trials conducted before 2000 would be indirect owing to the potential for patients to react differently to behavioural interventions. For example, mailed educational materials may be less effective in the digital age, in which physical mail is more quickly discarded. We did not, however, encounter interventions and outcomes dominated by evidence from before 2000, so we did not need to rate down the certainty for this reason.

We judged imprecision on the basis of the plausibility of the intervention achieving or exceeding the minimally important effect, without considering the possible magnitude of effect.^67^ We used minimally important differences, sourced from the literature and by consensus of the parallel BMJ Rapid Recommendations guideline panel to make these judgments. The final assessment of certainty was fully contextualised by the guideline panel to formulate recommendations.

To facilitate interpretation, we calculated the risk differences per 1000 patients for all dichotomous outcomes by multiplying the risk ratio obtained from meta-analyses with an estimate of the risk of the outcome in the control group. For the control group risk, we used the median risk reported in standard care groups across trials. The parallel BMJ Rapid Recommendations guideline panel provided input on the applicability of the control group risks.

We report results using GRADE simple language summaries (for example, describing high certainty evidence with declarative statements, moderate certainty evidence with “probably,” low certainty evidence with “may,” and very low certainty evidence with “very uncertain”).^68^ In the main manuscript, for comparisons and outcomes with multiple measures of the same outcome, we prioritise reporting results from the measure with the highest certainty. If certainty ratings are comparable, we report results from the most commonly used measure.

### Patient and public involvement

The parallel BMJ Rapid Recommendations guideline panel, which included three patient partners, provided input on the scope of the systematic review, including the outcomes of interest.

## Results

### Search results

**Fig 1:**
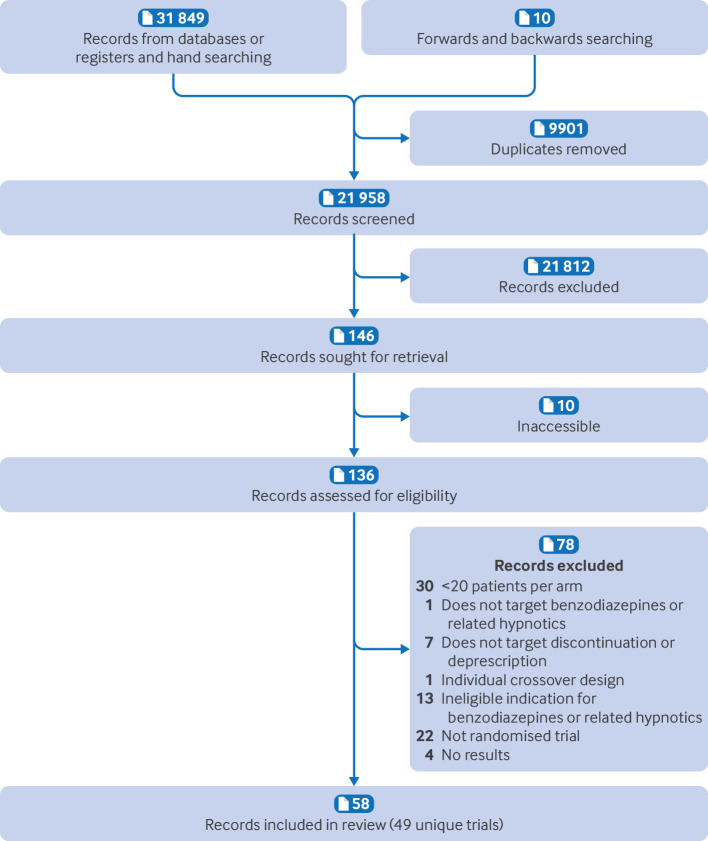
Study selection

Our search yielded more than 30 000 records. Of these, 58 publications, reporting on 49 unique trials with 39 336 patients, proved eligible. Eight trials were reported in more than one publication.^69^ ^70^ ^71^ ^72^ ^73^ ^74^ ^75^ ^76^ ^77^ ^78^ ^79^ ^80^ ^81^ ^82^ ^83^ ^84^ ^85^ Figure 1 shows study selection, and supplement 2 lists excluded studies and the reasons for exclusion. All trials were published in English.

### Trial and patient characteristics

Table 1 shows characteristics of trials and patients. Trials investigated a range of interventions, including tapering^73^ ^74^ ^78^ ^79^ ^82^ ^83^ ^84^ ^86^ ^87^ ^88^; education of patients^89^ ^90^ ^91^ ^92^ ^93^ ^94^ ^95^ ^96^ ^97^ ^98^; education of physicians^99^ ^100^ ^101^ ^102^ ^103^ ^104^ ^105^; the combination of education of patients and physicians^106^; cognitive behavioural therapy^71^ ^72^ ^76^ ^77^ ^78^ ^79^ ^84^ ^107^ ^108^ ^109^ ^110^ ^111^ ^112^ ^113^ ^114^; medication review^70^ ^81^ ^115^ ^116^; pharmacist led interventions including a multicomponent intervention involving pharmacists, a pharmacist led educational intervention, and the introduction of a clinical pharmacy service to nursing homes^117^ ^118^ ^119^; mindfulness and motivational interviewing^120^; drug assisted tapering and withdrawal^69^ ^75^ ^85^ ^88^ ^121^ ^122^ ^123^ ^124^ ^125^ ^126^; and auricular acupuncture.^110^ Trials typically compared active interventions against no additional intervention or a low intensity and minimally disruptive educational intervention targeting another topic or class of drugs other than insomnia and BSH (for example, antihypertensive agents), which we classified as usual care. Cognitive behavioural therapy, sometimes combined with tapering, was frequently compared against tapering alone, which we also classified as usual care in comparisons involving cognitive behavioural therapy.

**Table 1:** Characteristics of trials and participants. Values are numbers (percentages) unless stated otherwise

Characteristic	Trials (n=49)
Design:	
** **Parallel randomised trial	32 (65)
** **Cluster randomised trial	16 (33)
** **Crossover by cluster randomised trial	1 (2)
Country*:	
** **Argentina	1 (2)
** **Australia	3 (6)
** **Belgium	5 (10)
** **Brazil	1 (2)
** **Canada	8 (16)
** **Finland	2 (4)
** **Ireland	1 (2)
** **Israel	1 (2)
** **Japan	2 (4)
** **Netherlands	4 (8)
** **Spain	2 (4)
** **Sweden	3 (6)
** **Switzerland	1 (2)
** **United Kingdom	6 (12)
** **United States	11 (22)
** **Not reported	1 (2)
Registered	21 (43)
Funding*:	
** **Industry	7 (14)
** **Government	35 (71)
** **Institutional	11 (22)
** **Not-for-profit	11 (22)
** **None	2 (4)
Time published:	
** **1980-90	1 (2)
** **1991-2000	7 (14)
** **2001-10	19 (39)
** **2011-present	22 (45)
Median (IQR) duration of follow-up across studies, weeks	24 (14-50.5)
Percentage male across all trials	31.9
Weighted mean age, years	53.7
Median (IQR) No of participants	188 (70-528)
Targeted drugs:	
** **Benzodiazepines including or excluding other related hypnotics	39 (80)
** **Non-benzodiazepine hypnotics	3 (6)
** **Range of inappropriate drugs including benzodiazepines and related hypnotics	7 (14)
Interventions*	**No of trials (No of participants)**
Tapering	6 (1122)
Education of patients	10 (5570)
Education of physicians	7 (2291)
Education of patients and physicians	1 (2367)
Cognitive behavioural therapy	11 (1930)
Medication review	3 (2499)
Pharmacist led interventions	3 (23 117)
Mindfulness	1 (70)
Drug assisted tapering	9 (616)

IQR=interquartile range.

* A trial could be classified in more than one category.

Sixteen trials used a cluster randomised trial design,^82^ ^83^ ^96^ ^99^ ^100^ ^101^ ^103^ ^104^ ^105^ ^111^ ^117^ ^118^ ^119^ one a crossover by cluster design,^76^ ^77^ and the remainder parallel randomised designs. The median duration of follow-up was 24 (interquartile range 14-50.5) weeks. All trials were conducted in high income countries, apart from two trials conducted in Argentina and Brazil.^120^ ^121^ More than three quarters of trials were published since 2001. Four trials were conducted in nursing homes and the remainder in the community.^86^ ^100^ ^116^ ^118^ More than half of all trials were funded by government organisations, followed by not-for-profit organisations or hospitals and universities.

Three trials targeted non-benzodiazepine hypnotics,^69^ ^92^ ^110^ seven a range of inappropriate drugs including BSH,^95^ ^102^ ^104^ ^106^ ^115^ ^117^ ^118^ and the remainder benzodiazepines including or excluding other related hypnotics. Trials included a median of 188 (interquartile range 70-528) patients. Approximately one third of all patients were male. Twenty one trials with 5848 patients reported that most of their participants (>60%) were using BSH for insomnia.^69^ ^71^ ^72^ ^73^ ^74^ ^75^ ^76^ ^77^ ^82^ ^83^ ^85^ ^87^ ^90^ ^93^ ^96^ ^107^ ^108^ ^109^ ^110^ ^111^ ^112^ ^113^ ^114^ ^120^ ^121^ ^124^ The reasons for BSH use in other trials were not reported.

### Risk of bias

**Fig 2:**
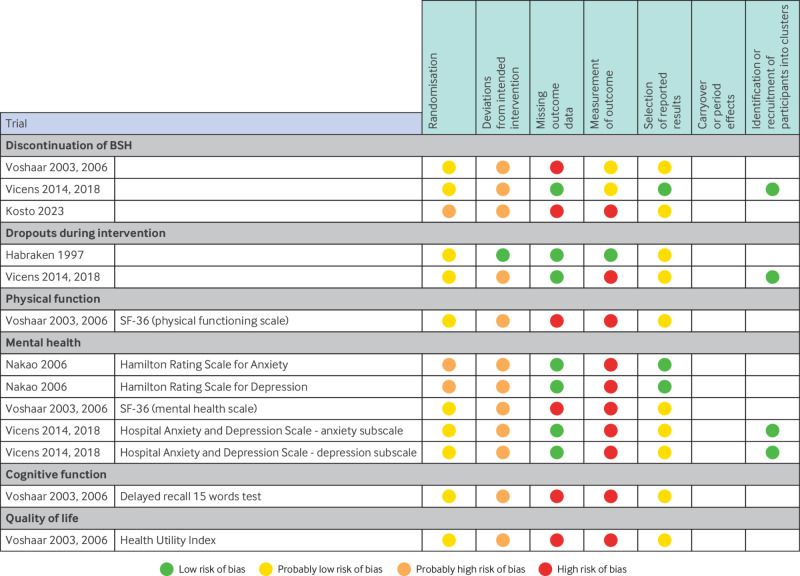
Risk of bias of trials that compared tapering with usual care

Figure 2 shows the risk of bias of trials that compared tapering with usual care, and supplements 3-10 show risk of bias judgments for all trials and outcomes. Nearly all data were rated as being at high risk of bias, primarily owing to concerns about deviations from intended intervention that arose from lack of masking of patients and healthcare providers and potential for differential care across trial arms. Concerns about the randomisation procedures and allocation concealment also contributed to ratings of high risk of bias.

### Summary of findings

**Fig 3:**
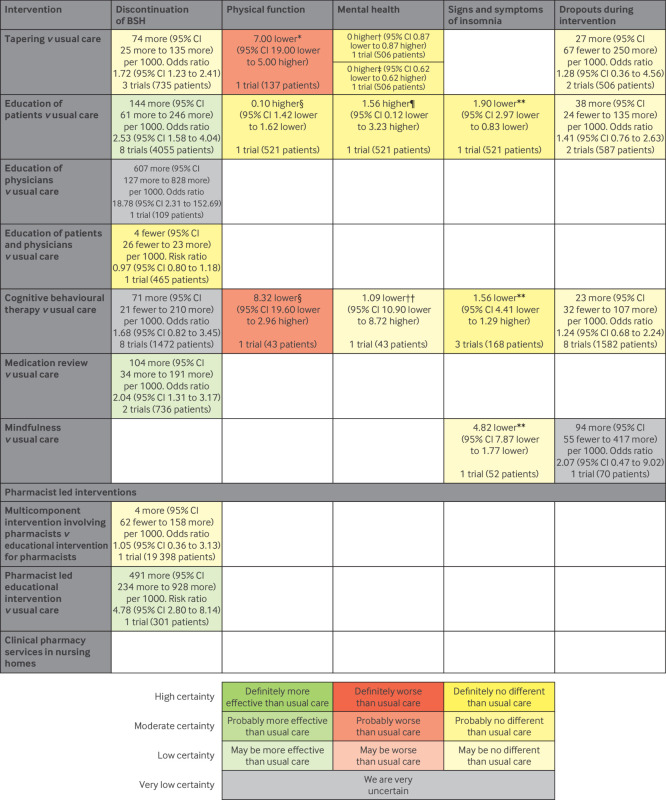
Summary of results of interventions excluding drug assisted tapering. *SF-36 physical functioning scale (range 0-100 with higher scores indicating better function). †Hospital Anxiety and Depression Scale anxiety subscale (range 0-21 with lower scores indicating better mental health). ‡Hospital Anxiety and Depression Scale depression subscale (range 0-21 with lower scores indicating better mental health). §SF-36 physical component score (range 0-100 with higher scores indicating better function). ¶SF-12 mental component score (range 0-100 with higher scores indicating better mental health). **Insomnia Severity Index (range 0-28 with lower scores indicating fewer signs and symptoms of insomnia). ††SF-36 mental component score (range 0-100 with higher scores indicating better mental health). CI=confidence interval

**Fig 4:**
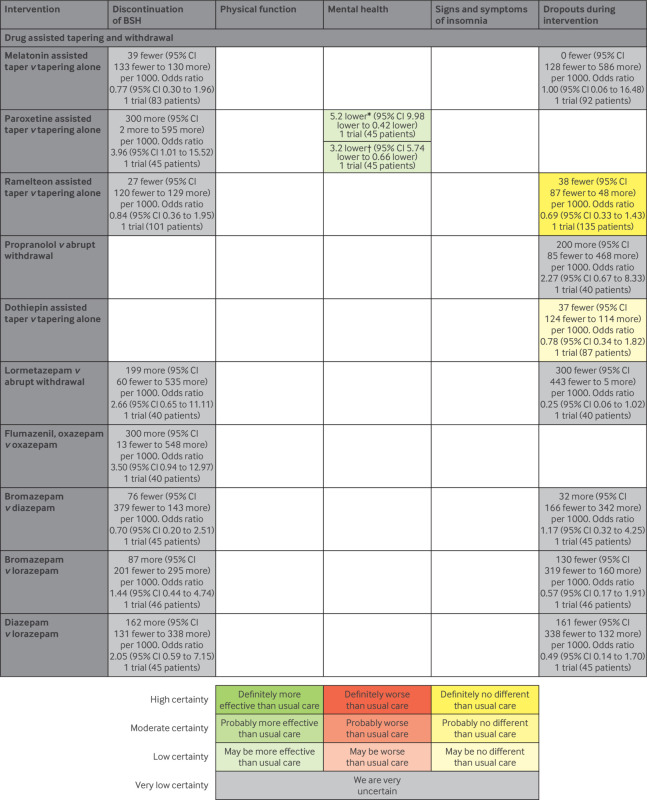
Summary of results for drug assisted tapering and withdrawal interventions. *Hamilton Rating Scale for anxiety (range: 0 to 56 with lower scores indicating less anxiety). †Hamilton Rating Scale for depression (range: 0 to 56 with lower scores indicating less depression). CI=confidence interval

Figure 3 and figure 4 summarise the results of this systematic review and meta-analysis. Low certainty evidence suggests that, compared with usual care, education of patients, medication review, and a pharmacist led educational intervention may increase the proportion of patients who discontinue BSH. However, we did not find evidence that these interventions improve physical function, mental health, or signs and symptoms of insomnia. Moderate certainty evidence suggests that education of patients probably has little or no effect on physical function, mental health, and signs and symptoms of insomnia. We did not find evidence assessing these outcomes for medication review or the pharmacist led educational intervention.

We did not find evidence that other interventions, including tapering, education of physicians, the combination of education of patients and physicians, cognitive behavioural therapy, mindfulness, other pharmacist led interventions, and drug assisted tapering or withdrawal may help patients to discontinue BSH. Moreover, we did not find high or moderate certainty evidence that any of the interventions caused an increase in dropouts. Finally, low certainty evidence suggests that multicomponent interventions may be more effective at facilitating deprescription of BSH than single component interventions. Below, we describe the results for each type of intervention.

#### Tapering

Six trials, with 1122 patients, reported on tapering.^73^ ^74^ ^78^ ^79^ ^82^ ^83^ ^84^ ^86^ ^87^ ^88^ Five trials, with 839 patients, compared the effects of tapering BSH against usual care.^82^ ^84^ ^86^ ^87^ ^88^ One trial compared two different tapering strategies, one with written instructions and one with follow-up appointments with physicians, against usual care.^82^ In this trial, patients were instructed to reduce their dose of BSH by 10-25% at two to three week intervals.^82^ A second trial reduced BSH by 25% at one week intervals for three weeks followed by 12.5% at one week intervals for two weeks.^86^ A third reduced BSH by 25% at two week intervals.^88^ The fourth trial reduced BSH by 25% per week with the option to divide the final step to 12.5% over four days.^84^ The final trial combined tapering with education.^87^ Patients in the tapering arm received information on insomnia, information on the harms of prolonged BSH use, sleep hygiene recommendations, a tapering instruction table, and a letter for their family physician requesting discontinuation of BSH.^87^ Patients in the usual care arm received only information on insomnia and the harms of BSH, along with the letter for their physician.^87^ This trial does not describe the pace of tapering. Supplement 11 shows additional trial characteristics, and supplement 12 shows the summary of findings table.

Low certainty evidence suggests that tapering may have little or no effect on discontinuation of BSH, dropouts during the intervention, and cognitive function, and may worsen quality of life and physical functioning. Although tapering seems to have a statistically significant effect on the proportion of patients who discontinue BSH, the absolute effect did not meet our threshold of clinical importance. Moderate certainty evidence suggests that tapering probably has little or no effect on symptoms of anxiety and depression.

We anticipated that the effects of tapering may differ according to the pace of tapering, reason for BSH use, and method for ascertaining discontinuation of BSH. We did not, however, find evidence of a difference in the effects of tapering based on pace or based on method for ascertaining discontinuation. Two trials reported including patients using BSH primarily for insomnia. An analysis restricted to these trials produced results that were consistent with the primary analysis.

The trial that compared written instructions for tapering against follow-up with physicians did not report a difference in discontinuation of BSH between the two tapering strategies.^82^ One trial compared masked tapering combined with an augmented cognitive behavioural therapy programme against unmasked tapering combined with standard cognitive behavioural therapy.^73^ ^74^ The trial suggests that masked tapering may increase the proportion of patients who discontinue BSH compared with unmasked tapering with cognitive behavioural therapy but may have little or no effect on signs and symptoms of insomnia compared with unmasked tapering.

#### Education of patients

Ten trials, with 5570 patients, investigated the effects of educating patients.^89^ ^90^ ^91^ ^92^ ^93^ ^94^ ^95^ ^96^ ^97^ ^98^ Nine trials, with 4709 patients, compared education of patients against usual care.^89^ ^90^ ^91^ ^92^ ^93^ ^94^ ^95^ ^96^ ^98^ Educational content described adverse effects of BSH and beneficial reasons for reducing BSH, tapering schedules, stories of peers who have successfully stopped or reduced BSH, self-assessment tools, and suggestions for alternative treatments to manage insomnia. One trial included educational materials that also had a link to a cognitive behavioural therapy website.^93^

Seven trials investigated the effects of single letters or distribution of written educational materials such as brochures,^89^ ^90^ ^91^ ^92^ ^93^ ^94^ ^96^ one trial investigated the effects of the distribution of multiple letters,^89^ four trials investigated the effects of written educational materials coupled with follow-up by physicians, pharmacists, or researchers,^91^ ^92^ ^93^ ^95^ and one trial investigated the effects of counselling by researchers or healthcare providers.^98^ Supplement 13 shows additional trial characteristics, and supplement 14 shows the summary of findings table.

Low certainty evidence suggests that education of patients may increase the proportion of patients who discontinue BSH and may have little or no effect on dropouts. Moderate certainty evidence also suggests that education of patients probably has little or no effect on physical function, mental health, signs and symptoms of insomnia, total sleep time, sleep onset latency, and sleep efficiency, although the results for these outcomes came from only a single trial and may not reflect the effects of all educational interventions targeting patients.^90^

We anticipated that the effect of educational interventions may differ according to the mode of the educational intervention, reason for BSH use, and method for ascertaining BSH discontinuation. We did not find evidence that the effect estimates varied according to the mode of the educational intervention or method for ascertaining BSH discontinuation. Three trials reported including patients using BSH primarily for insomnia.^90^ ^93^ ^96^ An analysis restricted to these trials produced results that were consistent with the primary analysis.

One trial compared a single generic letter, a single tailored letter, and three tailored letters.^97^ The results of the trial suggest that tailored letters may be more effective at achieving discontinuation than generic letters but did not report a difference between distributing a single tailored letter and three tailored letters. Another trial compared patient education through a passive letter or physician led consultations with patients against usual care.^91^ The trial did not report a statistically significant difference between the two education strategies. A third trial compared two educational materials that differed in their content and approach against usual care.^90^ The YAWNS-1 and YAWNS-2 packages were similar but had some differences. YAWNS-1 included a flexible dose reduction schedule and emphasised behaviour change techniques and cognitive behavioural therapy for insomnia. The YAWNS-2 package used a fixed dose reduction schedule, described risks of BSH, and described strategies for improving sleep. The results of the trial suggest that YAWNS-1 may be preferable to YAWNS-2.

#### Education of physicians

Seven trials, with more than 2000 patients, reported on interventions targeted at educating physicians.^99^ ^100^ ^101^ ^102^ ^103^ ^104^ ^105^ Six trials, with more than 2000 patients, compared education of physicians against usual care.^100^ ^101^ ^102^ ^103^ ^104^ ^105^ The educational interventions typically focused on the indications for BSH use, the effects of BSH in older people, strategies for counselling patients on reducing or discontinuing BSH, and alternative treatments for sleep. Some interventions focused on reducing physicians’ overall prescribing of BSH rather than educating physicians to encourage current users to discontinue BSH.

One trial reported on a multisession educational programme for nurses, nursing assistants, and physicians in nursing homes.^100^ Two trials reported on educational visits to physicians,^101^ ^102^ one of which also involved follow-up calls.^101^

Four trials investigated the combination of physician education with audit and feedback.^99^ ^103^ ^104^ ^105^ One trial compared four unique strategies: audit and feedback alone, audit and feedback combined with a letter from the medical regulatory authority body describing best practices and advice on how to reduce medications in patients receiving higher doses of BSH and links to external prescribing resources, audit and feedback combined with the letter and a phone call from a pharmacist, or audit and feedback combined with the letter and a phone call from a physician.^99^ Another trial compared audit and feedback combined with in-person education of physicians on doing medication reviews against usual care.^104^ This trial also provided incentive payments to physicians to do medication reviews.^104^ One trial compared audit and feedback combined with a two hour educational in-person workshop for physicians against usual care.^105^ The last trial compared sending written educational bulletins to physicians every two months for six months, also with audit and feedback, against usual care.^103^ Supplement 15 shows additional trial characteristics, and supplement 16 shows the summary of findings table.

Only one trial reported on discontinuation of BSH. Owing to problems of serious risk of bias and very serious imprecision, the evidence on the effects of education of physicians on the proportion of patients who discontinue BSH is very uncertain. Several trials reported the number of prescriptions or average dose of BSH in medical practices instead of discontinuation. These trials either reported no difference or a small reduction in prescriptions of BSH following education of physicians.^101^ ^102^ ^103^ ^104^ ^105^

We hypothesised that the effect of education of physicians may differ according to the educational strategy, reason for BSH use among patients, and method for ascertaining BSH discontinuation. Insufficient evidence precluded subgroup analyses to investigate these factors. The trial comparing audit and feedback—alone or combined with a letter from the medical regulatory authority or a phone call—did not report differences between the four educational strategies on the number of patients receiving high doses of BSH.^99^

#### Education of patients and physicians

One trial, with 2367 patients, compared an intervention that combined the education of patients and physicians against usual care.^106^ The trial targeted drugs acting on the central nervous system, including benzodiazepines, opioids, muscle relaxants, tricyclic antidepressants, and first generation antihistamines. Primary care practices within an integrated healthcare system were randomised to receive the intervention or usual care. Patient education comprised medication specific educational brochures, handouts with tips on managing symptoms with non-drug approaches, and a brochure on ways to prevent falls. Physician education comprised information on the target drug class, evidence based treatment alternatives, and practice support for deprescribing. Supplement 17 shows additional trial characteristics, and supplement 18 shows the summary of findings table.

Moderate certainty evidence suggests that a programme comprised of distributing educational materials to patients and healthcare providers probably has little or no effect on discontinuation of BSH compared with usual care. This comparison, however, was informed by a single trial, so the evidence may not be broadly applicable to all educational interventions targeting patients and physicians.

We hypothesised that the effect of education of patients and physicians may differ according to the educational strategy, reason for BSH use among patients, and method for ascertaining BSH discontinuation. Insufficient evidence precluded subgroup analyses to investigate these factors.

#### Cognitive behavioural therapy

Eleven trials, with 1930 patients, investigated cognitive behavioural therapy.^71^ ^72^ ^76^ ^77^ ^78^ ^79^ ^84^ ^107^ ^108^ ^109^ ^110^ ^111^ ^112^ ^113^ ^114^ Nine trials, with 1762 patients, compared cognitive behavioural therapy alone or in combination with tapering against usual care or tapering alone.^76^ ^84^ ^107^ ^108^ ^109^ ^111^ ^112^ ^113^ ^114^ Cognitive behavioural therapy typically involved principles of cognitive behavioural therapy for insomnia,^54^ such as education about stimulus control, sleep restriction, sleep hygiene, and dysfunctional beliefs about sleep. As most cognitive behavioural interventions included some suggestion of tapering, the analysis comparing cognitive behavioural therapy against usual care also includes trials that compared cognitive behavioural therapy in combination with tapering against tapering alone, trials that compared cognitive behavioural therapy in combination with tapering against no other interventions, and trials that compared cognitive behavioural therapy alone against tapering alone.

Four trials offered cognitive behavioural therapy in person and in groups,^84^ ^108^ ^113^ ^114^ three trials in person and individually,^76^ ^107^ ^112^ one trial using booklets,^109^ and another trial using an interactive e-learning program.^111^ The intensity of cognitive behavioural therapy varied from five sessions over 10 weeks to 10 sessions over 10 weeks. The duration of sessions varied between 50 minutes and two hours. Supplement 19 shows additional trial characteristics, and supplement 20 shows the summary of findings table.

Owing to concerns about risk of bias, inconsistency, and imprecision, we are very uncertain of the effects of cognitive behavioural therapy on the proportion of patients who discontinue BSH. Moderate certainty evidence suggests that cognitive behavioural therapy probably has little or no effect on signs and symptoms of insomnia, sleep onset latency, and sleep efficiency. Low certainty evidence also suggests that cognitive behavioural therapy may improve quality of life, may have little or no effect on dropouts, mental health, cognitive function, and total sleep time, and may worsen physical function.

We anticipated that the effects of cognitive behavioural therapy may differ according to whether it was administered alone or in combination with tapering, whether it was compared with no other intervention or tapering, the mode of delivery, the number of sessions, the reason for BSH use, and the method for ascertaining BSH discontinuation. We did subgroup analyses investigating the effects of these factors and did not find evidence that these factors influenced effect estimates.

One trial compared cognitive behavioural therapy against auricular acupuncture and did not find a statistically significant difference in discontinuation of BSH between the two groups.^110^ Another trial compared cognitive behavioural therapy alone or in combination with two different blinded tapering strategies (reduction of BSH by 10% or 20% every two weeks).^71^ More patients in the blinded tapering groups discontinued BSH than in the cognitive behavioural therapy alone group. Finally, a trial that compared cognitive behavioural therapy alone, cognitive behavioural therapy coupled with tapering, and tapering alone did not find a statistically significant difference between the three interventions in the number of patients that discontinued BSH.^113^

#### Medication review

Three trials, with 2499 patients, compared medication reviews with usual care.^70^ ^81^ ^115^ ^116^ In one trial, for older adults with multimorbidity and polypharmacy, a physician and a pharmacist jointly did a medication review supported by clinical decision software followed by shared decision making between the patient and physician.^70^ ^81^ In the second trial, the intervention was implemented in nursing homes and involved interdisciplinary case conferences with the general practitioner, pharmacist, and nurse doing medication reviews for each nursing home resident.^116^ In this trial, medication review was also combined with education of healthcare providers, which included e-learning and a face-to-face workshop covering pharmacotherapy for older people. The last trial implemented a medication review, done by pharmacists and nurses, for home healthcare patients.^115^ Supplement 21 shows additional trial characteristics, and supplement 22 shows the summary of findings table.

Low certainty evidence suggests that medication review may increase the proportion of patients who discontinue BSH. We hypothesised that the effect of medication review may differ according to the reason for BSH use among patients and the method for ascertaining BSH discontinuation. Insufficient evidence precluded subgroup analyses to investigate these factors.

#### Mindfulness intervention

One trial, with 70 patients, compared eight sessions of a group mindfulness based relapse prevention intervention coupled with motivational interviewing against motivational interviewing alone.^120^ Supplement 23 shows additional trial characteristics, and supplement 24 shows the summary of findings table.

We are very uncertain of the effects of mindfulness on dropouts during intervention. Low certainty evidence suggests that mindfulness may have little or no effect on signs and symptoms of insomnia.

We anticipated that the effects of mindfulness based interventions may differ according to the mode of delivery, number of sessions, reason for BSH use, and method for ascertaining BSH discontinuation. Insufficient evidence, however, precluded subgroup analyses based on these factors.

#### Pharmacist led interventions

Three trials, with 23 117 patients, reported on pharmacist led interventions.^117^ ^118^ ^119^ Supplement 25 shows additional trial characteristics, and supplements 26-28 show summary of findings tables.

One of these trials compared an intervention that encouraged pharmacists to distribute educational materials to patients and physicians against usual care.^117^ Materials for patients included drug specific brochures outlining why the drug might be inappropriate, alternative treatment options, and a visual tapering protocol. Materials for physicians provided the rationale for deprescribing, evidence on drug harms, credible sources for recommendations, and safer alternatives. Low certainty evidence suggests that this intervention may increase the proportion of patients who discontinue BSH.

The second trial compared an intensive support programme in which pharmacists received training to work with physicians to identify long term BSH users and send letters encouraging patients to discontinue BSH against a programme in which pharmacists received a manual to perform the same task.^119^ Low certainty evidence suggests that the intensive programme may have little or no effect on BSH discontinuation.

The last trial investigated the effects of introducing clinical pharmacy services in nursing homes compared with usual care.^118^ The trial reported moderate certainty evidence that the clinical pharmacy service probably reduced the number of prescriptions for benzodiazepines in nursing homes compared with usual care.

Insufficient evidence precluded any subgroup or sensitivity analyses.

#### Drug assisted tapering or withdrawal

Nine trials, with 616 patients, reported on drug assisted tapering or withdrawal.^69^ ^75^ ^85^ ^88^ ^121^ ^122^ ^123^ ^124^ ^125^ ^126^ Two trials, with 140 patients, compared melatonin assisted tapering against tapering without melatonin^75^ ^121^; one trial, with 66 patients, compared tapering with paroxetine against tapering alone and usual care^88^; one trial, with 135 patients, compared tapering with ramelteon against tapering alone^69^; one trial, with 40 patients, compared abrupt withdrawal with propranolol against abrupt withdrawal alone^126^; and one trial, with 87 patients, compared tapering with dothiepin against tapering alone.^125^

Three trials involved replacing patients’ regular BSH with an alternative BSH or placebo.^122^ ^123^ ^124^ In the first trial, 40 patients’ BSH was replaced with either lormetazepam or placebo for one week, after which the drug was discontinued.^124^ In another trial, 40 patients were randomised to either discontinue BSH and receive oxazepam for the first three nights coupled with flumazenil for eight nights or receive oxazepam for the first three nights without flumazenil.^122^ In the last trial, 68 patients were randomised to be switched to diazepam, lorazepam, or bromazepam, after which each of the drugs was tapered over 10 weeks.^123^ Supplement 29 shows additional trial characteristics, and supplements 30-39 show summary of findings tables.

Tapering with paroxetine compared with tapering alone may improve anxiety and depression, but the evidence is of low certainty. The evidence on the effects of tapering with melatonin compared with tapering alone, tapering with paroxetine compared with tapering alone, tapering with ramelteon compared with tapering alone, switching to lormetazepam before withdrawal compared with withdrawal alone, flumazenil and oxazepam supported withdrawal compared with oxazepam alone, and switching to bromazepam, lorazepam, or diazepam before withdrawal on discontinuation of BSH is very uncertain. Insufficient evidence precluded subgroup analyses.

#### Multicomponent interventions

Eleven trials, with 20 250 patients, compared multicomponent with single component interventions.^71^ ^72^ ^76^ ^77^ ^78^ ^79^ ^84^ ^99^ ^107^ ^108^ ^109^ ^112^ ^113^ ^119^ ^120^ Supplement 40 shows the summary of findings table. Low certainty evidence suggests that multicomponent interventions may increase the proportion of patients who discontinue BSH compared with single component interventions. We did not find compelling evidence that multicomponent interventions led to more dropouts during the intervention.

## Discussion

### Main findings

Our systematic review and meta-analysis summarised all the evidence from randomised trials assessing the effectiveness of interventions to discontinue or facilitate deprescription of BSH in healthcare settings. We reviewed 49 unique randomised trials, with more than 30 000 patients, and found that education of patients, medication reviews, and a pharmacist led educational intervention may increase the proportion of patients who discontinue BSH.

We did not find evidence that other interventions increase the proportion of patients who discontinue BSH, including tapering, education of physicians, the combination of education of patients and physicians, cognitive behavioural therapy, mindfulness, other pharmacist led interventions, or drug assisted tapering or withdrawal. The evidence for these interventions was either very low certainty or did not suggest an important effect.

### Interpretation of findings

The current body of evidence is overall uncertain, primarily because of concerns about risk of bias and imprecision. For many interventions, such as medication reviews and pharmacist led interventions, no evidence is available on several outcomes important to patients, including physical function, mental health, and signs and symptoms of insomnia. Trials also seldom reported outcomes beyond one year of follow-up. Initial successes in discontinuing BSH might not be sustained in the long term.

Many of the interventions described in this review can be implemented in various ways. For example, tapering strategies can vary in the degree of follow-up by healthcare providers and the pace of tapering. We had insufficient evidence to confidently conclude whether the effects of interventions may vary according to their specific characteristics. Even for comparisons for which we were able to do subgroup analyses to investigate the influence of other intervention characteristics on effect estimates, the effect estimates within subgroups were imprecise.

Our results may not generalise to all patients. Fewer than half of trials reported most patients to be predominantly using BSH for insomnia. Whether the effects of interventions for deprescribing BSH may be different depending on the reason for its use is unclear. Moreover, the evidence came primarily from patients in their 50s, despite the use of BSH being highly prevalent and likely more problematic in older adults. Nearly all trials excluded patients with significant mental health conditions, so whether these results also apply to those with comorbid mental health diagnoses is uncertain. Poor reporting and insufficient evidence also precluded investigation of how factors such as the habitual dose of BSH, degree of dependence, and patient’s adherence may influence the effects of interventions.

Additionally, the effects of interventions facilitated by healthcare providers may vary depending on healthcare providers’ knowledge, skills, motivation, and self-efficacy. Healthcare providers who choose to participate in clinical trials may be more knowledgeable and skilled than others and may hold stronger beliefs about the effectiveness of the interventions that are under study.

Not all interventions to reduce BSH may be feasible across all health systems. Many trials examined costly, time intensive interventions that overburdened health systems may struggle to support. For instance, although pharmacist led education of patients and physicians seemed promising, pharmacists’ scope and nature of practice varies across jurisdictions and settings and is often constrained by limited time and high workloads. Health systems may mitigate this challenge by offering incentives to support the implementation of such interventions.

We are uncertain of the optimal triggers and contraindications for BSH deprescription. Patients who enrol in clinical trials and adhere to the intervention may be more willing to discontinue BSH than are those in the general population. However, this willingness likely applies equally to patients randomised to the control group, making it unlikely to meaningfully bias our results.

Finally, key organisational aspects of interventions that may contribute to their success remain uninvestigated. Except for one trial that offered financial incentives to physicians for doing medication reviews,^104^ the effectiveness of changes in financial and governance structures has not been studied. These factors, along with implementation strategies and the context in which interventions are implemented, may affect their success.

### Relation to previous literature and guidance

This systematic review builds on previous reviews by providing the most up-to-date evidence. Previous reviews have been restricted to select interventions,^37^ ^39^ ^44^ ^45^ ^127^ have not adequately differentiated between various types of interventions,^18^ ^44^ have been restricted to older adults,^37^ ^127^ have not provided quantitative synthesis of study results,^18^ ^40^ ^45^ or have not assessed the certainty of the body of evidence.^37^ ^39^ ^40^ ^45^ This review adheres to the highest established standards for producing trustworthy, transparent, and actionable systematic reviews: we assessed the risk of bias of studies, provided quantitative estimates of the effects of interventions, and evaluated the certainty of evidence by using the latest GRADE guidance.^128^ Our findings are largely consistent with other reviews. We show that although certain interventions may be effective in increasing the proportion of patients who discontinue BSH, the evidence is overall of low certainty.

Our findings on the effectiveness of tapering contradicts existing guidance that recommends tapering.^129^ We show that although tapering increases the number of patients who discontinue BSH, this effect is modest and may not be clinically important. This may partly be attributed to the effects of tapering alone not being sustained in the long term. In our review, we prioritised extracting and synthesising effects at the longest reported point of follow-up. Trials typically reported a reduction in the effects of tapering over time.^78^ ^79^ ^82^ ^83^ ^84^

Our findings on the effectiveness of cognitive behavioural therapy also contrast with those of a previous review that found it to be effective.^45^ This discrepancy can be attributed to differences in eligibility criteria and the time point of interest. For example, a previous review reports results for the most positive time point for a trial investigating cognitive behavioural therapy instead of the longest reported point of follow-up.^113^ At the longest reported point of follow-up, the data suggest that initial cessation of BSH was not later sustained.

### Strengths and limitations of study

The strengths of our systematic review and meta-analysis include a rigorous and comprehensive search for eligible randomised trials, screening and extraction of data in duplicate, the application of GRADE methods to evaluate the certainty of evidence, and our focus on outcomes important to patients.

Despite our rigorous search of the literature, we might have missed eligible trials, particularly publications in languages other than English. We mitigated this problem by also reviewing the references of similar systematic reviews and soliciting experts about eligible trials that may not have come up in our search.^37^ ^38^ ^39^ ^40^ ^41^ ^42^ ^43^ ^44^ ^45^ We assessed the certainty of evidence by using the GRADE approach. Although the GRADE approach presents a comprehensive framework for systematically and transparently considering all factors that may bear on the certainty of evidence, its application is ultimately subjective, and other authors may come to different conclusions about the certainty of evidence.^130^ We anticipated that the effects of interventions may vary according to several characteristics of patients and interventions such as age and duration of BSH use, but we were unable to do subgroup analyses owing to insufficient data. A group of content experts and methodologists, with feedback from the wider guideline panel, classified similar interventions together for synthesis. In making these classifications, we considered the objective of the intervention, the target stakeholder, the class of therapy, and whether the intervention involved gradual or abrupt reduction in BSH dose, educational materials, or meetings with professionals. Other researchers might classify the interventions differently.

### Implications of findings

This systematic review and meta-analysis presents a range of interventions that may be useful in reducing prolonged use of BSH. We found the body of evidence to be overall of low certainty, highlighting the need for additional research.

Many professional societies and authoritative organisations advise against the prolonged use of BSH, citing evidence suggesting that these drugs are associated with adverse events.^1^ ^2^ ^18^ This systematic review informs parallel BMJ Rapid Recommendations issued by the BE-SAFE consortium. Its dedicated panel appraised this evidence to issue a conditional recommendation in favour of deprescription.^34^

Decision makers also need to consider practitioners’ workload.^48^ Although certain interventions may be effective, some of these interventions may reduce the amount of time that healthcare providers have available for other medical problems and so may not be feasible in overburdened settings. We also found other interventions that could be implemented with little disruption to existing healthcare services and workflows. Such interventions, including sending letters and booklets to patients, are unlikely to significantly affect the workload of healthcare providers.

Future trials that investigate the effects of pragmatic interventions (that is, those that could be implemented in the real world without massive interference with other healthcare services or demanding unrealistic resources or time from healthcare providers) are still needed. These trials should recruit sufficient participants to allow for more precise effect estimates and use sufficient safeguards against bias. For example, as the nature of the interventions will likely preclude masking of patients and healthcare providers, investigators may still reduce potential for bias by matching interventions with regard to the degree of interaction between patients and healthcare providers to reduce expectancy effects and use strict treatment and follow-up protocols to reduce deviations from the intended intervention.

### Conclusion

The evidence to guide patients and clinicians on the effectiveness of strategies to discontinue BSH is uncertain. Low certainty evidence suggests that educating patients, medication review, and enabling pharmacists to educate patient and physicians may increase the proportion of patients who discontinue BSH. We did not find compelling evidence that tapering, education of physicians, the combination of education of patients and physicians, cognitive behavioural therapy, mindfulness, other pharmacist led interventions, and drug supported tapering and withdrawal strategies are effective. Finally, low certainty evidence suggests that multicomponent interventions may be more effective at facilitating discontinuation of BSH than single component interventions.

## What is already known on this topic

Many patients use benzodiazepines and related sedative hypnotics (BSH) to treat insomnia despite risks such as falls, cognitive impairment, and dependence
Healthcare providers have reported limited knowledge of alternatives to BSH, a lack of clarity on its harms, and insufficient evidence on optimal strategies to support its discontinuation

## What this study adds

Low certainty evidence suggests that patient education, medication reviews, and pharmacist led educational interventions may increase BSH discontinuation rates
Multicomponent interventions may be more effective than single component approaches, but further high quality research is needed
This low certainty body of evidence highlights the need for additional high quality research

## Data Availability

All data are available on Open Science Framework (https://osf.io/8em4p/files/osfstorage).

## Web extra

Extra material supplied by authors

Web appendix: Supplementary materials

## Notes

Contributors: DZ, TA, SKN, AS, AC, SVV, and SS conceived the study. RC developed the search strategy. ML, TJ, SK, TP, TA, SVV, SS, JPL, and SKN screened studies for eligibility and collected data. DZ and TP did analyses. DZ wrote the first draft of the manuscript. All co-authors interpreted the data, substantively contributed to the manuscript, and reviewed and approved the final draft of the manuscript. TA supervised the study. DZ and TA are the guarantors. The corresponding author attests that all listed authors meet authorship criteria and that no others meeting the criteria have been omitted.

Transparency: The lead authors (the manuscript’s guarantors) affirm that the manuscript is an honest, accurate, and transparent account of the study being reported; that no important aspects of the study have been omitted; and that any discrepancies from the study as planned (and, if relevant, registered) have been explained.

Dissemination to participants and related patient and public communities: This systematic review is intended to inform forthcoming BMJ Rapid Recommendations.

Provenance and peer review: Not commissioned; externally peer reviewed.
