# Supplementary material for: Comparative effectiveness of interventions to facilitate deprescription of benzodiazepines and other sedative hypnotics: systematic review and meta-analysis
Source: BMJ. 2025 Jun 17;389:e081336. doi: 10.1136/bmj-2024-081336 (PMC12171951; doi:10.1136/bmj-2024-081336)
Supplement: Supplementary file 1 — Web appendix: Supplementary materials [file zerd081336.ww1.pdf]

The comparative effectiveness of interventions to facilitate discontinuation of benzodiazepines and other sedative hypnotics: A systematic review and meta-analysis

Table of Contents

The comparative effectiveness of interventions to facilitate discontinuation of benzodiazepines and other sedative hypnotics: A systematic review and meta-analysis 1

Supplement 1: Search Strategy 4

Supplement 2: Excluded studies and reasons for exclusion 17

Supplement 3: Risk of bias of trials that compared education of patients with usual care 26

Supplement 4: Risk of bias of trials that compared education of physicians against usual care 27

Supplement 5: Risk of bias of trials that compared education of patients and physicians against usual care 28

Supplement 6: Risk of bias of trials that compared cognitive behavioral therapy (CBT) against usual care 29

Supplement 7: Risk of bias of trials that compared medication review against usual care 30

Supplement 8: Risk of bias of trials that compared mindfulness against usual care 31

Supplement 9: Risk of bias of trials that addressed pharmacist-led interventions 32

Supplement 10: Risk of bias of trials that addressed pharmacologic-assisted tapering or abrupt withdrawal 33

Supplement 11: Table of trial characteristics for trials that addressed tapering 34

Supplement 12: Summary of findings for the comparison of tapering against usual care 36

Supplement 13: Table of trial characteristics for trials that investigated education of patients 37

Supplement 14: Summary of findings for the comparison of education of patients against usual care ... 42

Supplement 15: Table of trial characteristics for trials that investigated education of physicians 43

Supplement 16: Summary of findings for the comparison of education of physicians against usual care 47

Supplement 17: Table of trial characteristics for trials that investigated education of patients and physicians 48

Supplement 18: Summary of findings for the comparison of education of patients and physicians against usual care 49

Supplement 19: Table of trial characteristics for trials that investigated cognitive behavioral therapy ... 50

Supplement 20: Summary of findings for the comparison of CBT against usual care 55

Supplement 21: Table of trial characteristics for trials that investigated medication review 57

Supplement 22: Summary of findings for the comparison of medication review against usual care 59

Supplement 23: Table of trial characteristics for trials that investigated mindfulness 60

Supplement 24: Summary of findings for the comparison of mindfulness against motivational interviewing 61

Supplement 25: Table of trial characteristics for trials that investigated pharmacist-led interventions... 62

Supplement 26: Summary of findings for the comparison of pharmacist-led educational intervention against usual care 64
Supplement 27: Summary of findings for the comparison of a multicomponent intervention involving pharmacists against an educational intervention for pharmacists 65
Supplement 28: Summary of findings for the comparison of the introduction of clinical pharmacy services in nursing homes against usual care 66
Supplement 29: Table of trial characteristics for trials that investigated pharmacologic-assisted tapering 67
Supplement 30: Summary of findings for the comparison of tapering with melatonin against tapering alone 70
Supplement 31: Summary of findings for the comparison of tapering with paroxetine against tapering alone 71
Supplement 32: Summary of findings for the comparison of tapering with ramelteon against tapering alone 72
Supplement 33: Summary of findings for the comparison of tapering with dothiepin against tapering alone 73
Supplement 34: Summary of findings for the comparison of tapering with flumazenil and oxazepam against oxazepam alone 74
Supplement 35: Summary of findings for the comparison of abrupt withdrawal with propranolol against abrupt withdrawal alone 75
Supplement 36: Summary of findings for the comparison of switching to lormetazepam before abrupt withdrawal against abrupt withdrawal alone 76
Supplement 37: Summary of findings for the comparison of switching to bromazepam before tapering against switching to diazepam before tapering 77
Supplement 38: Summary of findings for the comparison of switching to bromazepam before tapering against switching to lorazepam before tapering 78
Supplement 39: Summary of findings for the comparison of switching to diazepam before tapering against switching to lorazepam before tapering 79
Supplement 40: Summary of findings for the comparison of multicomponent interventions against single component interventions 80

Supplement 1: Search Strategy
MEDLINE

Database: OVID Medline Epub Ahead of Print, In-Process & Other Non-Indexed Citations, Ovid MEDLINE(R) Daily and Ovid MEDLINE(R) 1946 to Present
Search Strategy:
----
1 exp Benzodiazepines/ (69094)
2 (Benzodiazepine* or BZD* or Bromazepam or anxyrex or apo-bromazepam or bromalich or bromaz 1a pharma or bromazanil or bromazep or durazanil or lexatin or lexomil or lexotan or lexotanil or Alprazolam or alprazolan or xanor or alprox or alpraz or apo-alpraz or cassadan or esparon or kalma or alprazol or novo-alprazol or nu alpraz or nu-alpraz or ralozam or tafil or trankimazin or xanax or Bromazepam or anxyrex or bromazepam or apo-bromazepam or bromazepam or bromalich or bromaz or bromazanil or bromazep or bromazepam or durazanil or lexatin or lexomil or lexotan or lexotanil or Chlordiazepoxide or chlozepid or elenium or librium or methaminodiazepoxide or Clobazam or perizam or tapclob or zacco or frisium or onfi or urbanyl).mp. [mp=title, book title, abstract, original title, name of substance word, subject heading word, floating sub-heading word, keyword heading word, organism supplementary concept word, protocol supplementary concept word, rare disease supplementary concept word, unique identifier, synonyms] (55484)
3 (Clonazepam or klonopin or antelepsin or rivotril or Clorazepate or tranxene or chlorazepate or clorazepic or tranxilium or Diazepam or valium or apaurin or diazemuls or faustan or relanium or seduxen or sibazon or stesolid or Estazolam or nuctalon or prosom or tasedan or flunitrazepam or flunibeta or flunimerck or fluninoc or flunizep or fluridrazepam or narcozep or rohipnol or rohypnol or Flurazepam or apo-flurazepam or dalmadorm or dalmane or dormodor or staurodorm or Halazepam or paxipam or alapryl or pacinone or Ketazolam or anxon or anseren or ansieten or ansietil or atenual or loftran or marcen or sedatival or sedotime or solatran or unakalm or marcen or Loprazolam or dormonoc).mp. [mp=title, book title, abstract, original title, name of substance word, subject heading word, floating sub-heading word, keyword heading word, organism supplementary concept word, protocol supplementary concept word, rare disease supplementary concept word, unique identifier, synonyms] (35184)
4 (Lorazepam or apo-lorazepam or Ativan or donix or duralozam or durazolam or idalprem or laubeel or lorazep or lorazem or novo-lorazem or nu loraz or nu-loraz or orfidal or sedicepan or sinestron or somagerol or temesta or tavor or tolid or orfida or Lormetazepam or noctamid or methyllorazepam or n-methyllorazepam or noctamid or loramet or Medazepam or nobrium or rudotel or rusedal or Nitrazepam or alodorm or dormalon or dormo-puren or eatan or imadorm or imeson or mogadon or nitrazadon or nitrazep or nitrodiazepam or novanox or radedorm or remnos or rhoxal-nitrazepam or serenade or somnite).mp. [mp=title, book title, abstract, original title, name of substance word, subject heading word, floating sub-heading word, keyword heading word, organism supplementary concept word, protocol supplementary concept word, rare disease supplementary concept word, unique identifier, synonyms] (6609)
5 (Nordazepam or calmday or dealkylprazepam or demethyldiazepam or deoxydemoxepam or desmethyldiazepam or n desalkylhalazepam or n descyclopropylmethyl prazepam or n descyclopropylmethylprazepam or n destrifluoroethylhalazepam or n-desalkylhalazepam or n-descyclopropylmethyl-prazepam or n-descyclopropylmethylprazepam or n-destrifluoroethylhalazepam or nordaz or nordiazepam or norprazepam or tranxilium n or vegesan or Oxazepam or serenid or

serepax or seresta or adumbran or serax or tazepam or Prazepam or centrax or demetrin or lysanxia or mono demetrin or prazepam or reapam).mp. [mp=title, book title, abstract, original title, name of substance word, subject heading word, floating sub-heading word, keyword heading word, organism supplementary concept word, protocol supplementary concept word, rare disease supplementary concept word, unique identifier, synonyms] (2834)
6 (Quazepam or doral or prosedar or quazium or dormalin or oniria or quazapam or cetrane or quiedorm or Temazepam or hydroxydiazepam or 3-hydroxydiazepam or apo-temazepam or dasuen or euhypnos or gen-temazepam or levanxol or methyloxazepam or nocturne or norkotral tema or normison or normitab or nortem or oxydiazepam or planum or pronervon or remestan or restoril or signopam or norkotral or temaze or temazep or temtabs or tenox or Triazolam or apo-triazo or halcion or trilam).mp. [mp=title, book title, abstract, original title, name of substance word, subject heading word, floating sub-heading word, keyword heading word, organism supplementary concept word, protocol supplementary concept word, rare disease supplementary concept word, unique identifier, synonyms] (4712)
7 or/1-6 (98105)
8 deprescriptions/ (933)
9 (Deprescri* or De-prescri* or Unprescri* or Discontin* or Cease* or Ceasing* or Cessation* or Withdraw* or Reduc* or taper*).mp. [mp=title, book title, abstract, original title, name of substance word, subject heading word, floating sub-heading word, keyword heading word, organism supplementary concept word, protocol supplementary concept word, rare disease supplementary concept word, unique identifier, synonyms] (4532745)
10 8 or 9 (4532745)
11 7 and 10 (23809)
12 randomized controlled trial.pt. (585496)
13 controlled clinical trial.pt. (95170)
14 randomi?ed.ab. (707319)
15 placebo.ab. (235601)
16 drug therapy.fs. (2566948)
17 randomly.ab. (401591)
18 trial.ab. (635109)
19 groups.ab. (2474815)
20 or/12-19 (5600061)
21 exp animals/ not humans.sh. (5087714)
22 20 not 21 (4886312)
23 11 and 22 (9961)

Embase (OVID)
Database: Embase <1974 to 2023 January 30>
Search Strategy:
----
1 exp benzodiazepine/ (30795)
2 (Benzodiazepine* or BZD* or Bromazepam or anxyrex or apo-bromazepam or bromalich or bromaz 1a pharma or bromazanil or bromazep or durazanil or lexatin or lexomil or lexotan or lexotanil or Alprazolam or alprazolan or xanor or alprox or alpraz or apo-alpraz or cassadan or esparon or kalma or

alprazol or novo-alprazol or nu alpraz or nu-alpraz or ralozam or tafil or trankimazin or xanax or Bromazepam or anxyrex or bromazepam or apo-bromazepam or bromazepam or bromalich or bromaz or bromazanil or bromazep or bromazepam or durazanil or lexatin or lexomil or lexotan or lexotanil or Chlordiazepoxide or chlozepid or elenium or librium or methaminodiazepoxide or Clobazam or perizam or tapclob or zacco or frisium or onfi or urbanyl).mp. [mp=title, abstract, heading word, drug trade name, original title, device manufacturer, drug manufacturer, device trade name, keyword heading word, floating subheading word, candidate term word] (131708)

3 (Clonazepam or klonopin or antelepsin or rivotril or Clorazepate or tranxene or chlorazepate or clorazepic or tranxilium or Diazepam or valium or apaurin or diazemuls or faustan or relanium or seduxen or sibazon or stesolid or Estazolam or nuctalon or prosom or tasedan or flunitrazepam or flunibeta or flunimerck or fluninoc or flunizep or fluridrazepam or narcozep or rohipnol or rohypnol or Flurazepam or apo-flurazepam or dalmadorm or dalmane or dormodor or staurodorm or Halazepam or paxipam or alapryl or pacinone or Ketazolam or anxon or anseren or ansieten or ansietil or atenual or loftran or marcen or sedatival or sedotime or solatran or unakalm or marcen or Loprazolam or dormonoc).mp. [mp=title, abstract, heading word, drug trade name, original title, device manufacturer, drug manufacturer, device trade name, keyword heading word, floating subheading word, candidate term word] (110672)

4 (Lorazepam or apo-lorazepam or Ativan or donix or duralozam or durazolam or idalprem or laubeel or lorazep or lorazem or novo-lorazem or nu loraz or nu-loraz or orfidal or sedicepan or sinestron or somagerol or temesta or tavor or tolid or orfida or Lormetazepam or noctamid or methyllorazepam or n-methyllorazepam or noctamid or loramet or Medazepam or nobrium or rudotel or rusedal or Nitrazepam or alodorm or dormalon or dormo-puren or eatan or imadorm or imeson or mogadon or nitrazadon or nitrazep or nitrodiazepam or novanox or radedorm or remnos or rhoxal-nitrazepam or serenade or somnite).mp. [mp=title, abstract, heading word, drug trade name, original title, device manufacturer, drug manufacturer, device trade name, keyword heading word, floating subheading word, candidate term word] (36751)

5 (Nordazepam or calmday or dealkylprazepam or demethyldiazepam or deoxydemoxepam or desmethyldiazepam or n desalkylhalazepam or n descyclopropylmethyl prazepam or n descyclopropylmethylprazepam or n destrifluoroethylhalazepam or n-desalkylhalazepam or n-descyclopropylmethyl-prazepam or n-descyclopropylmethylprazepam or n-destrifluoroethylhalazepam or nordaz or nordiazepam or norprazepam or tranxilium n or vegesan or Oxazepam or serenid or serepax or seresta or adumbran or serax or tazepam or Prazepam or centrax or demetrin or lysanxia or mono demetrin or prazepam or reapam).mp. [mp=title, abstract, heading word, drug trade name, original title, device manufacturer, drug manufacturer, device trade name, keyword heading word, floating subheading word, candidate term word] (10715)

6 (Quazepam or doral or prosedar or quazium or dormalin or oniria or quazapam or cetrane or quiedorm or Temazepam or hydroxydiazepam or 3-hydroxydiazepam or apo-temazepam or dasuen or euhypnos or gen-temazepam or levanxol or methyloxazepam or nocturne or norkotral tema or normison or normitab or nortem or oxydiazepam or planum or pronervon or remestan or restoril or signopam or norkotral or temaze or temazep or temtabs or tenox or Triazolam or apo-triazo or halcion or trilam).mp. [mp=title, abstract, heading word, drug trade name, original title, device manufacturer, drug manufacturer, device trade name, keyword heading word, floating subheading word, candidate term word] (13369)

7 or/1-6 (229040)

8 deprescription/ (1275)
9 (Deprescri* or De-prescri* or Unprescri* or Discontin* or Cease* or Ceasing* or Cessation* or Withdraw* or Reduc* or taper*).mp. (6335614)
10 8 or 9 (6335614)
11 7 and 10 (60242)
12 randomized controlled trial/ (759166)
13 Controlled clinical study/ (467823)
14 random$.ti,ab. (1900430)
15 randomization/ (97632)
16 intermethod comparison/ (289614)
17 placebo.ti,ab. (356911)
18 (compare or compared or comparison).ti. (588568)
19 ((evaluated or evaluate or evaluating or assessed or assess) and (compare or compared or comparing or comparison)).ab. (2664322)
20 (open adj label).ti,ab. (104871)
21 ((double or single or doubly or singly) adj (blind or blinded or blindly)).ti,ab. (267934)
22 double blind procedure/ (204739)
23 parallel group$1.ti,ab. (31161)
24 (crossover or cross over).ti,ab. (121321)
25 ((assign$ or match or matched or allocation) adj5 (alternate or group$1 or intervention$1 or patient$1 or subject$1 or participant$1)).ti,ab. (401780)
26 (assigned or allocated).ti,ab. (473328)
27 (controlled adj7 (study or design or trial)).ti,ab. (434785)
28 (volunteer or volunteers).ti,ab. (276626)
29 human experiment/ (626784)
30 trial.ti. (386682)
31 or/12-30 (6106484)
32 (random$ adj sampl$ adj7 ("cross section$" or questionnaire$1 or survey$ or database$1)).ti,ab. not (comparative study/ or controlled study/ or randomi?ed controlled.ti,ab. or randomly assigned.ti,ab.) (9233)
33 Cross-sectional study/ not (randomized controlled trial/ or controlled clinical study/ or controlled study/ or randomi?ed controlled.ti,ab. or control group$1.ti,ab.) (328185)
34 (((case adj control$) and random$) not randomi?ed controlled).ti,ab. (20860)
35 (Systematic review not (trial or study)).ti. (240027)
36 (nonrandom$ not random$).ti,ab. (18476)
37 "Random field$".ti,ab. (2849)
38 (random cluster adj3 sampl$).ti,ab. (1496)
39 (review.ab. and review.pt.) not trial.ti. (1060026)
40 "we searched".ab. and (review.ti. or review.pt.) (46591)
41 "update review".ab. (134)
42 (databases adj4 searched).ab. (57652)
43 (rat or rats or mouse or mice or swine or porcine or murine or sheep or lambs or pigs or piglets or rabbit or rabbits or cat or cats or dog or dogs or cattle or bovine or monkey or monkeys or trout or marmoset$1).ti. and animal experiment/ (1201583)

44 Animal experiment/ not (human experiment/ or human/) (2523155)
45 or/32-44 (4184633)
46 31 not 45 (5397878)
47 11 and 46 (11833)

PsycInfo (OVID)

Database: APA PsycInfo <1806 to January Week 4 2023>

Search Strategy:

----

1 exp benzodiazepines/ (11246)

2 (Benzodiazepine* or BZD* or Bromazepam or anxyrex or apo-bromazepam or bromalich or bromaz 1a pharma or bromazanil or bromazep or durazanil or lexatin or lexomil or lexotan or lexotanil or Alprazolam or alprazolan or xanor or alprox or alpraz or apo-alpraz or cassadan or esparon or kalma or alprazol or novo-alprazol or nu alpraz or nu-alpraz or ralozam or tafil or trankimazin or xanax or Bromazepam or anxyrex or bromazepam or apo-bromazepam or bromazepam or bromalich or bromaz or bromazanil or bromazep or bromazepam or durazanil or lexatin or lexomil or lexotan or lexotanil or Chlordiazepoxide or chlozepid or elenium or librium or methaminodiazepoxide or Clobazam or perizam or tapclob or zacco or frisium or onfi or urbanyl).mp. [mp=title, abstract, heading word, table of contents, key concepts, original title, tests & measures, mesh word] (19470)

3 (Clonazepam or klonopin or antelepsin or rivotril or Clorazepate or tranxene or chlorazepate or clorazepic or tranxilium or Diazepam or valium or apaurin or diazemuls or faustan or relanium or seduxen or sibazon or stesolid or Estazolam or nuctalon or prosom or tasedan or flunitrazepam or flunibeta or flunimerck or fluninoc or flunizep or fluridrazepam or narcozep or rohipnol or rohypnol or Flurazepam or apo-flurazepam or dalmadorm or dalmane or dormodor or staurodorm or Halazepam or paxipam or alapryl or pacinone or Ketazolam or anxon or anseren or ansieten or ansietil or atenual or loftran or marcen or sedatival or sedotime or solatran or unakalm or marcen or Loprazolam or dormonoc).mp. [mp=title, abstract, heading word, table of contents, key concepts, original title, tests & measures, mesh word] (7630)

4 (Lorazepam or apo-lorazepam or Ativan or donix or duralozam or durazolam or idalprem or laubeel or lorazep or lorazem or novo-lorazem or nu loraz or nu-loraz or orfidal or sedicepan or sinestron or somagerol or temesta or tavor or tolid or orfida or Lormetazepam or noctamid or methyllorazepam or n-methyllorazepam or noctamid or loramet or Medazepam or nobrium or rudotel or rusedal or Nitrazepam or alodorm or dormalon or dormo-puren or eatan or imadorm or imeson or mogadon or nitrazadon or nitrazep or nitrodiazepam or novanox or radedorm or remnos or rhoxal-nitrazepam or serenade or somnite).mp. [mp=title, abstract, heading word, table of contents, key concepts, original title, tests & measures, mesh word] (2298)

5 (Nordazepam or calmday or dealkylprazepam or demethyldiazepam or deoxydemoxepam or desmethyldiazepam or n desalkylhalazepam or n descyclopropylmethyl prazepam or n

descyclopropylmethylprazepam or n destrifluoroethylhalazepam or n-desalkylhalazepam or n-descyclopropylmethyl-prazepam or n-descyclopropylmethylprazepam or n-destrifluoroethylhalazepam or nordaz or nordiazepam or norprazepam or tranxilium n or vegesan or Oxazepam or serenid or serepax or seresta or adumbran or serax or tazepam or Prazepam or centrax or demetrin or lysanxia or mono demetrin or prazepam or reapam).mp. [mp=title, abstract, heading word, table of contents, key concepts, original title, tests & measures, mesh word] (507)

6 (Quazepam or doral or prosedar or quazium or dormalin or oniria or quazapam or cetrane or quiedorm or Temazepam or hydroxydiazepam or 3-hydroxydiazepam or apo-temazepam or dasuen or euhypnos or gen-temazepam or levanxol or methyloxazepam or nocturne or norkotral tema or normison or normitab or nortem or oxydiazepam or planum or pronervon or remestan or restoril or signopam or norkotral or temaze or temazep or temtabs or tenox or Triazolam or apo-triazo or halcion or trilam).mp. [mp=title, abstract, heading word, table of contents, key concepts, original title, tests & measures, mesh word] (1408)

7 or/1-6 (26256)

8 (Deprescri* or De-prescri* or Unprescri* or Discontin* or Cease* or Ceasing* or Cessation* or Withdraw* or Reduc* or taper*).mp. [mp=title, abstract, heading word, table of contents, key concepts, original title, tests & measures, mesh word] (579178)

9 7 and 8 (8448)

10 random:.tw. or placebo:.mp. or double-blind:.tw. (261856)

11 ((treatment or control) adj3 group*).ab. (121335)

12 (allocat* adj5 group*).ab. (3069)

13 ((clinical or control*) adj3 trial).mp. [mp=title, abstract, heading word, table of contents, key concepts, original title, tests & measures, mesh word] (54208)

14 or/10-13 (353916)

15 9 and 14 (1847)

CINAHL (EBSCO

Tuesday, January 31, 2023 9:19:49 PM				
#	**Query**	**Limiters/Expanders**	**Last Run Via**	**Results**
S35	S31 AND S34	Search modes - Boolean/Phrase	Interface - EBSCOhost Research Databases<br>Search Screen - Advanced Search<br>Database - CINAHL	1,455
S34	S32 OR S33	Search modes - Boolean/Phrase	Interface - EBSCOhost Research Databases<br>Search Screen - Advanced Search<br>Database - CINAHL	823,372
S33	TX Deprescri* or De-	Search modes -	Interface - EBSCOhost	823,37

	prescri* or Unprescri* or Discontin* or Cease* or Ceasing* or Cessation* or Withdraw* or Reduc* or taper*	Boolean/Phrase	Research Databases<br>Search Screen - Advanced Search<br>Database - CINAHL	2
S32	(MH "Deprescribing")	Search modes - Boolean/Phrase	Interface - EBSCOhost<br>Research Databases<br>Search Screen - Advanced Search<br>Database - CINAHL	236
S31	S22 AND S30	Search modes - Boolean/Phrase	Interface - EBSCOhost<br>Research Databases<br>Search Screen - Advanced Search<br>Database - CINAHL	4,044
S30	S24 OR S25 OR S26 OR S27 OR S28 OR S29	Search modes - Boolean/Phrase	Interface - EBSCOhost<br>Research Databases<br>Search Screen - Advanced Search<br>Database - CINAHL	17,841
S29	TX Quazepam or doral or prosedar or quazium or dormalin or oniria or quazapam or cetrane or quiedorm or Temazepam or hydroxydiazepam or 3-hydroxydiazepam or apo-temazepam or dasuen or euhypnos or gen-temazepam or levanxol or methyloxazepam or nocturne or norkotral tema or normison or normitab or nortem or oxydiazepam or planum or pronervon or remestan or restoril or signopam or norkotral or temaze or temazep or temtabs or tenox or Triazolam or apo-triazo or halcion or trilam	Search modes - Boolean/Phrase	Interface - EBSCOhost<br>Research Databases<br>Search Screen - Advanced Search<br>Database - CINAHL	780
S28	TX Nordazepam or calmday or dealkylprazepam or demethyldiazepam or deoxydemoxepam or desmethyldiazepam or n desalkylhalazepam or n descyclopropylmethyl prazepam or n descyclopropylmethylprazepam or n destrifluoroethylhalazep	Search modes - Boolean/Phrase	Interface - EBSCOhost<br>Research Databases<br>Search Screen - Advanced Search<br>Database - CINAHL	160

	am or n-desalkylhalazepam or n-descyclopropylmethyl-prazepam or n-descyclopropylmethylprazepam or n-destrifluoroethylhalazepam or nordaz or nordiazepam or norprazepam or tranxilium n or vegesan or Oxazepam or serenid or serepax or seresta or adumbran or serax or tazepam or Prazepam or centrax or demetrin or lysanxia or mono demetrin or prazepam or reapam			
S27	TX Lorazepam or apo-lorazepam or Ativan or donix or duralozam or durazolam or idalprem or laubeel or lorazep or lorazem or novo-lorazem or nu loraz or nu-loraz or orfidal or sedicepan or sinestron or somagerol or temesta or tavor or tolid or orfida or Lormetazepam or noctamid or methyllorazepam or n-methyllorazepam or noctamid or loramet or Medazepam or nobrium or rudotel or rusedal or Nitrazepam or alodorm or dormalon or dormo-puren or eatan or imadorm or imeson or mogadon or nitrazadon or nitrazep or nitrodiazepam or novanox or radedorm or remnos or rhoxal-nitrazepam or serenade or somnite	Search modes - Boolean/Phrase	Interface - EBSCOhost Research Databases<br>Search Screen - Advanced Search<br>Database - CINAHL	1,682
S26	TX Clonazepam or klonopin or antelepsin or rivotril or Clorazepate or tranxene or chlorazepate or clorazepic or tranxilium	Search modes - Boolean/Phrase	Interface - EBSCOhost Research Databases<br>Search Screen - Advanced Search<br>Database - CINAHL	3,289

	or Diazepam or valium or apaurin or diazemuls or faustan or relanium or seduxen or sibazon or stesolid or Estazolam or nuctalon or prosom or tasedan or flunitrazepam or flunibeta or flunimerck or fluninoc or flunizep or fluridrazepam or narcozep or rohipnol or rohypnol or Flurazepam or apo-flurazepam or dalmadorm or dalmane or dormodor or staurodorm or Halazepam or paxipam or alapryl or pacinone or Ketazolam or anxon or anseren or ansieten or ansietil or atenual or loftran or marcen or sedatival or sedotime or solatran or unakalm or marcen or Loprazolam or dormonoc			
S25	TX Benzodiazepine* or BZD* or Bromazepam or anxyrex or apo-bromazepam or bromalich or bromaz 1a pharma or bromazanil or bromazep or durazanil or lexatin or lexomil or lexotan or lexotanil or Alprazolam or alprazolan or xanor or alprox or alpraz or apo-alpraz or cassadan or esparon or kalma or alprazol or novo-alprazol or nu alpraz or nu-alpraz or ralozam or tafil or trankimazin or xanax or Bromazepam or anxyrex or bromazepam or apo-bromazepam or bromazepam or bromalich or bromaz or bromazanil or bromazep or bromazepam or durazanil or lexatin or lexomil or lexotan or	Search modes - Boolean/Phrase	Interface - EBSCOhost Research Databases<br>Search Screen - Advanced Search<br>Database - CINAHL	11,350

	lexotanil or Chlordiazepoxide or chlozepid or elenium or librium or methaminodiazepoxide or Clobazam or perizam or tapclob or zacco or frisium or onfi or urbanyl			
S24	(MH "Antianxiety Agents, Benzodiazepine+")	Search modes - Boolean/Phrase	Interface - EBSCOhost Research Databases<br>Search Screen - Advanced Search<br>Database - CINAHL	11,066
S23	S22 NOT S21	Expanders - Apply equivalent subjects<br>Search modes - Boolean/Phrase	Interface - EBSCOhost Research Databases<br>Search Screen - Advanced Search<br>Database - CINAHL	947,990
S22	S1 OR S2 OR S3 OR S4 OR S5 OR S6 OR S7 OR S8 OR S9 OR S10 OR S11 OR S12 OR S13 OR S14 OR S15	Expanders - Apply equivalent subjects<br>Search modes - Boolean/Phrase	Interface - EBSCOhost Research Databases<br>Search Screen - Advanced Search<br>Database - CINAHL	994,863
S21	S19 NOT S20	Expanders - Apply equivalent subjects<br>Search modes - Boolean/Phrase	Interface - EBSCOhost Research Databases<br>Search Screen - Advanced Search<br>Database - CINAHL	211,678
S20	MH (human)	Expanders - Apply equivalent subjects<br>Search modes - Boolean/Phrase	Interface - EBSCOhost Research Databases<br>Search Screen - Advanced Search<br>Database - CINAHL	2,637,603
S19	S16 OR S17 OR S18	Expanders - Apply equivalent subjects<br>Search modes - Boolean/Phrase	Interface - EBSCOhost Research Databases<br>Search Screen - Advanced Search<br>Database - CINAHL	245,318
S18	TI (animal model*)	Expanders - Apply equivalent subjects<br>Search modes - Boolean/Phrase	Interface - EBSCOhost Research Databases<br>Search Screen - Advanced Search<br>Database - CINAHL	3,436
S17	MH (animal studies)	Expanders - Apply equivalent subjects<br>Search modes - Boolean/Phrase	Interface - EBSCOhost Research Databases<br>Search Screen - Advanced Search<br>Database - CINAHL	150,488
S16	MH animals+	Expanders - Apply equivalent subjects<br>Search modes - Boolean/Phrase	Interface - EBSCOhost Research Databases<br>Search Screen - Advanced Search<br>Database - CINAHL	103,796
S15	AB (cluster W3 RCT)	Expanders - Apply	Interface - EBSCOhost	485

		equivalent subjects<br>Search modes - Boolean/Phrase	Research Databases<br>Search Screen - Advanced Search<br>Database - CINAHL	
S14	MH (crossover design) OR MH (comparative studies)	Expanders - Apply equivalent subjects<br>Search modes - Boolean/Phrase	Interface - EBSCOhost Research Databases<br>Search Screen - Advanced Search<br>Database - CINAHL	468,704
S13	AB (control W5 group)	Expanders - Apply equivalent subjects<br>Search modes - Boolean/Phrase	Interface - EBSCOhost Research Databases<br>Search Screen - Advanced Search<br>Database - CINAHL	141,643
S12	PT (randomized controlled trial)	Expanders - Apply equivalent subjects<br>Search modes - Boolean/Phrase	Interface - EBSCOhost Research Databases<br>Search Screen - Advanced Search<br>Database - CINAHL	148,991
S11	MH (placebos)	Expanders - Apply equivalent subjects<br>Search modes - Boolean/Phrase	Interface - EBSCOhost Research Databases<br>Search Screen - Advanced Search<br>Database - CINAHL	13,580
S10	MH (sample size) AND AB (assigned OR allocated OR control)	Expanders - Apply equivalent subjects<br>Search modes - Boolean/Phrase	Interface - EBSCOhost Research Databases<br>Search Screen - Advanced Search<br>Database - CINAHL	4,439
S9	TI (trial)	Expanders - Apply equivalent subjects<br>Search modes - Boolean/Phrase	Interface - EBSCOhost Research Databases<br>Search Screen - Advanced Search<br>Database - CINAHL	175,163
S8	AB (random*)	Expanders - Apply equivalent subjects<br>Search modes - Boolean/Phrase	Interface - EBSCOhost Research Databases<br>Search Screen - Advanced Search<br>Database - CINAHL	392,192
S7	TI (randomised OR randomized)	Expanders - Apply equivalent subjects<br>Search modes - Boolean/Phrase	Interface - EBSCOhost Research Databases<br>Search Screen - Advanced Search<br>Database - CINAHL	136,169
S6	MH cluster sample	Expanders - Apply equivalent subjects<br>Search modes - Boolean/Phrase	Interface - EBSCOhost Research Databases<br>Search Screen - Advanced Search<br>Database - CINAHL	5,189
S5	MH pretest-posttest design	Expanders - Apply equivalent subjects<br>Search modes - Boolean/Phrase	Interface - EBSCOhost Research Databases<br>Search Screen - Advanced Search<br>Database - CINAHL	51,761
S4	MH random assignment	Expanders - Apply equivalent subjects	Interface - EBSCOhost Research Databases	77,538

		Search modes - Boolean/Phrase	Search Screen - Advanced Search<br>Database - CINAHL	
S3	MH single-blind studies	Expanders - Apply equivalent subjects<br>Search modes - Boolean/Phrase	Interface - EBSCOhost Research Databases<br>Search Screen - Advanced Search<br>Database - CINAHL	15,890
S2	MH double-blind studies	Expanders - Apply equivalent subjects<br>Search modes - Boolean/Phrase	Interface - EBSCOhost Research Databases<br>Search Screen - Advanced Search<br>Database - CINAHL	53,861
S1	MH randomized controlled trials	Expanders - Apply equivalent subjects<br>Search modes - Boolean/Phrase	Interface - EBSCOhost Research Databases<br>Search Screen - Advanced Search<br>Database - CINAHL	135,766

Cochrane Library (Wiley)

Search Name:
Date Run: 31/01/2023 22:58:56
Comment:

ID Search Hits
#1 MeSH descriptor: [Benzodiazepines] explode all trees 10496
#2 Benzodiazepine* or BZD* or Bromazepam or anxyrex or apo-bromazepam or bromalich or bromaz 1a pharma or bromazanil or bromazep or durazanil or lexatin or lexomil or lexotan or lexotanil or Alprazolam or alprazolan or xanor or alprox or alpraz or apo-alpraz or cassadan or esparon or kalma or alprazol or novo-alprazol or nu alpraz or nu-alpraz or ralozam or tafil or trankimazin or xanax or Bromazepam or anxyrex or bromazepam or apo-bromazepam or bromazepam or bromalich or bromaz or bromazanil or bromazep or bromazepam or durazanil or lexatin or lexomil or lexotan or lexotanil or Chlordiazepoxide or chlozepid or elenium or librium or methaminodiazepoxide or Clobazam or perizam or tapclob or zacco or frisium or onfi or urbanyl 8709
#3 Clonazepam or klonopin or antelepsin or rivotril or Clorazepate or tranxene or chlorazepate or clorazepic or tranxilium or Diazepam or valium or apaurin or diazemuls or faustan or relanium or seduxen or sibazon or stesolid or Estazolam or nuctalon or prosom or tasedan or flunitrazepam or flunibeta or flunimerck or fluninoc or flunizep or fluridrazepam or narcozep or rohipnol or rohypnol or Flurazepam or apo-flurazepam or dalmadorm or dalmane or dormodor or staurodorm or Halazepam or paxipam or alapryl or pacinone or Ketazolam or anxon or anseren or ansieten or ansietil or atenual or loftran or marcen or sedatival or sedotime or solatran or unakalm or marcen or Loprazolam or dormonoc 6117
#4 Lorazepam or apo-lorazepam or Ativan or donix or duralozam or durazolam or idalprem or laubeel or lorazep or lorazem or novo-lorazem or nu loraz or nu-loraz or orfidal or sedicepan or sinestron or somagerol or temesta or tavor or tolid or orfida or Lormetazepam or noctamid or

methyllorazepam or n-methyllorazepam or noctamid or loramet or Medazepam or nobrium or rudotel or rusedal or Nitrazepam or alodorm or dormalon or dormo-puren or eatan or imadorm or imeson or mogadon or nitrazadon or nitrazep or nitrodiazepam or novanox or radedorm or remnos or rhoxal-nitrazepam or serenade or somnite 2720

#5 Nordazepam or calmday or dealkylprazepam or demethyldiazepam or deoxydemoxepam or desmethyldiazepam or n desalkylhalazepam or n descyclopropylmethyl prazepam or n descyclopropylmethylprazepam or n destrifluoroethylhalazepam or n-desalkylhalazepam or n-descyclopropylmethyl-prazepam or n-descyclopropylmethylprazepam or n-destrifluoroethylhalazepam or nordaz or nordiazepam or norprazepam or tranxilium n or vegesan or Oxazepam or serenid or serepax or seresta or adumbran or serax or tazepam or Prazepam or centrax or demetrin or lysanxia or mono demetrin or prazepam or reapam 642

#6 Quazepam or doral or prosedar or quazium or dormalin or oniria or quazapam or cetrane or quiedorm or Temazepam or hydroxydiazepam or apo-temazepam or dasuen or euhypnos or levanxol or methyloxazepam or nocturne or normison or normitab or nortem or oxydiazepam or planum or pronervon or remestan or restoril or signopam or norkotral or temaze or temazep or temtabs or tenox or Triazolam or apo-triazo or halcion or trilam 1427

#7 #2 or #3 or #4 or #5 or #6 15709

#8 MeSH descriptor: [Deprescriptions] explode all trees 68

#9 Deprescri* or De-prescri* or Unprescri* or Discontin* or Cease* or Ceasing* or Cessation* or Withdraw* or Reduc* or taper* 557626

#10 #8 or #9 557627

#11 #7 and #10 in Trials 4975

Supplement 2: Excluded studies and reasons for exclusion

Reason for Exclusion	Number of Studies	Study
Not RCT	22	Ashton H. Benzodiazepine withdrawal: outcome in 50 patients. Br J Addict. 1987;82(6):665-71.<br><br>Bashir K, King M, Ashworth M. Controlled evaluation of brief intervention by general practitioners to reduce chronic use of benzodiazepines. Br J Gen Pract. 1994;44(386):408-12.<br><br>Bergman H, Borg S, Engelbrektson K, Vikander B. Dependence on sedative-hypnotics: neuropsychological impairment, field dependence and clinical course in a 5-year follow-up study. Br J Addict. 1989;84(5):547-53.<br><br>Bernik MA, Gorenstein C, Vieira Filho AH. Stressful reactions and panic attacks induced by flumazenil in chronic benzodiazepine users. J Psychopharmacol. 1998;12(2):146-50.<br><br>Cadogan CA. Pharmacist interventions to deprescribe benzodiazepines in older adults: A missed opportunity? Res Social Adm Pharm. 2022;18(11):4012-5.<br><br>Christiaens A, Aubert CE, Wichniak A, Salvà Casanova A, Spinewine A. Deprescribing benzodiazepine receptor agonists for insomnia in older adults. Lancet. 2023;402(10411):1421-2.<br><br>Espie CA, Lindsay WR, Brooks DN. Substituting behavioural treatment for drugs in the treatment of insomnia: an exploratory study. J Behav Ther Exp Psychiatry. 1988;19(1):51-6.<br><br>Gilhooly TC, Webster MG, Poole NW, Ross S. What happens when doctors stop prescribing temazepam? Use of alternative therapies. Br J Gen Pract. 1998;48(434):1601-2.<br><br>Higgitt AC, Lader MH, Fonagy P. Clinical management of benzodiazepine dependence. Br Med J (Clin Res Ed). 1985;291(6497):688-90.<br><br>Kirmil-Gray K, Eagleston JR, Thoresen CE, Zarcone VP, Jr. Brief consultation and stress management treatments for drug-dependent insomnia: effects on sleep quality,

		self-efficacy, and daytime stress. J Behav Med. 1985;8(1):79-99.<br><br>Lader MH, Morton SV. A pilot study of the effects of flumazenil on symptoms persisting after benzodiazepine withdrawal. J Psychopharmacol. 1992;6(3):357-63.<br><br>Morin CM, Colecchi CA, Ling WD, Sood RK. Cognitive behavior therapy to facilitate benzodiazepine discontinuation among hypnotic-dependent patients with insomnia. Behavior Therapy. 1995;26(4):733-45.<br><br>Nishimura M, Teo AR, Mochizuki T, Fujiwara N, Nakamura M, Yamashita D. Feasibility and perceptions of a benzodiazepine deprescribing quality improvement initiative for primary care providers in Japan. BMC Prim Care. 2024;25:35.<br><br>Petrovic M, Pevernagie D, Van Den Noortgate N, Mariman A, Michielsen W, Afschrift M. A programme for short-term withdrawal from benzodiazepines in geriatric hospital inpatients: success rate and effect on subjective sleep quality. Int J Geriatr Psychiatry. 1999;14(9):754-60.<br><br>Ray WA, Taylor JA, Meador KG, Lichtenstein MJ, Griffin MR, Fought R, et al. Reducing antipsychotic drug use in nursing homes. A controlled trial of provider education. Arch Intern Med. 1993;153(6):713-21.<br><br>Savic I, Widen L, Stone-Elander S. Feasibility of reversing benzodiazepine tolerance with flumazenil. Lancet. 1991;337(8734):133-7.<br><br>Schweizer E, Case WG, Rickels K. Benzodiazepine dependence and withdrawal in elderly patients. Am J Psychiatry. 1989;146(4):529-31.<br><br>Shorr RI, Robin DW. Rational use of benzodiazepines in the elderly. Drugs Aging. 1994;4(1):9-20.<br><br>Spielman AJ, Saskin P, Thorpy MJ. Treatment of chronic insomnia by restriction of time in bed. Sleep. 1987;10(1):45-56.<br><br>Tattersall ML, Hallstrom C. Self-help and benzodiazepine withdrawal. J Affect Disord. 1992;24(3):193-8.<br><br>Tyrer P. Clinical management of benzodiazepine

		dependence. Br Med J (Clin Res Ed). 1985;291(6507):1507.<br><br>Zavesicka L, Brunovsky M, Matousek M, Sos P. Discontinuation of hypnotics during cognitive behavioural therapy for insomnia. BMC Psychiatry. 2008;8:80.
Individual crossover design	1	Peles E, Hetzroni T, Bar-Hamburger R, Adelson M, Schreiber S. Melatonin for perceived sleep disturbances associated with benzodiazepine withdrawal among patients in methadone maintenance treatment: a double-blind randomized clinical trial. Addiction. 2007;102(12):1947-53.
Does not target benzodiazepines/related hypnotics	1	Avorn J, Soumerai SB. Improving drug-therapy decisions through educational outreach. A randomized controlled trial of academically based "detailing". N Engl J Med. 1983;308(24):1457-63.
Does not target discontinuation/deprescription	7	Allain H, Coz FL, Borderies P, Schuck S, Giclais BdL, Patat AA, Gandon JM. Use of zolpidem 10 mg as a benzodiazepine substitute in 84 patients with insomnia. Human Psychopharmacology: Clinical and Experimental. 1998;13.<br><br>Curran HV, Collins R, Fletcher S, Kee SC, Woods B, Iliffe S. Older adults and withdrawal from benzodiazepine hypnotics in general practice: effects on cognitive function, sleep, mood and quality of life. Psychol Med. 2003;33(7):1223-37.<br><br>Lemoine P, Allain H, Janus C, Sutet P. Gradual withdrawal of zopiclone (7.5 mg) and zolpidem (10 mg) in insomniacs treated for at least 3 months. Eur Psychiatry. 1995;10 Suppl 3:161s-5s.<br><br>Lichstein KL, Johnson RS. Relaxation for insomnia and hypnotic medication use in older women. Psychol Aging. 1993;8(1):103-11.<br><br>Salzman C, Fisher J, Nobel K, Glassman R, Wolfson A, Kelley M. Cognitive improvement following benzodiazepine discontinuation in elderly nursing home residents. International Journal of Geriatric Psychiatry. 1992;7(2):89-93.<br><br>Shapiro C, Sherman D, Peck D. Withdrawal from benzodiazepines by initially switching to zopiclone. Eur Psychiatry. 1995;10 Suppl 3:145s-51s.

		Sharma GS, Saini LK, Mohan L, Kulshreshtha P, Dhamija P, Rao R, Kundu K, Gupta R. Yoga therapy versus cognitive behavioral therapy for chronic insomnia (CBT-I) – A randomized equivalence trial, an interim analysis. Sleep Med. 2024;115(Suppl 1):S196.
Ineligible indication for benzodiazepines/related hypnotics	13	Gosselin P, Ladouceur R, Morin CM, Dugas MJ, Baillargeon L. Benzodiazepine discontinuation among adults with GAD: A randomized trial of cognitive-behavioral therapy. J Consult Clin Psychol. 2006;74(5):908-19.<br><br>Hadley SJ, Mandel FS, Schweizer E. Switching from long-term benzodiazepine therapy to pregabalin in patients with generalized anxiety disorder: a double-blind, placebo-controlled trial. J Psychopharmacol. 2012;26(4):461-70.<br><br>Klein E, Colin V, Stolk J, Lenox RH. Alprazolam withdrawal in patients with panic disorder and generalized anxiety disorder: vulnerability and effect of carbamazepine. Am J Psychiatry. 1994;151(12):1760-6.<br><br>Lemoine P, Kermadi I, Garcia-Acosta S, Garay RP, Dib M. Double-blind, comparative study of cyamemazine vs. bromazepam in the benzodiazepine withdrawal syndrome. Prog Neuropsychopharmacol Biol Psychiatry. 2006;30(1):131-7.<br><br>Mercier-Guyon C, Chabannes JP, Saviuc P. The role of captodiamine in the withdrawal from long-term benzodiazepine treatment. Curr Med Res Opin. 2004;20(9):1347-55.<br><br>O'Connor K, Marchand A, Brousseau L, Aardema F, Mainguy N, Landry P, et al. Cognitive-behavioural, pharmacological and psychosocial predictors of outcome during tapered discontinuation of benzodiazepine. Clin Psychol Psychother. 2008;15(1):1-14.<br><br>Otto MW, Pollack MH, Sachs GS, Reiter SR, Meltzer-Brody S, Rosenbaum JF. Discontinuation of benzodiazepine treatment: efficacy of cognitive-behavioral therapy for patients with panic disorder. Am J Psychiatry. 1993;150(10):1485-90.<br><br>Rickels K, DeMartinis N, García-España F, Greenblatt DJ, Mandos LA, Rynn M. Imipramine and buspirone in treatment of patients with generalized anxiety disorder

		who are discontinuing long-term benzodiazepine therapy. Am J Psychiatry. 2000;157(12):1973-9.<br><br>Rickels K, Downing R, Schweizer E, Hassman H. Antidepressants for the treatment of generalized anxiety disorder. A placebo-controlled comparison of imipramine, trazodone, and diazepam. Arch Gen Psychiatry. 1993;50(11):884-95.<br><br>Romach MK, Kaplan HL, Busto UE, Somer G, Sellers EM. A controlled trial of ondansetron, a 5-HT3 antagonist, in benzodiazepine discontinuation. J Clin Psychopharmacol. 1998;18(2):121-31.<br><br>Vicens C, Fiol F, Llobera J, Campoamor F, Mateu C, Alegret S, Socias I. Withdrawal from long-term benzodiazepine use: randomised trial in family practice. Br J Gen Pract. 2006;56(533):958-63.]<br><br>Vorma H, Naukkarinen H, Sarna S, Kuoppasalmi K. Treatment of out-patients with complicated benzodiazepine dependence: comparison of two approaches. Addiction. 2002;97(7):851-9.<br><br>Zitman FG, Couvee JE. Chronic benzodiazepine use in general practice patients with depression: an evaluation of controlled treatment and taper-off: report on behalf of the Dutch Chronic Benzodiazepine Working Group. Br J Psychiatry. 2001;178:317-24.
<20 patients/arm	30	Ashton CH, Rawlins MD, Tyrer SP. A double-blind placebo-controlled study of buspirone in diazepam withdrawal in chronic benzodiazepine users. Br J Psychiatry. 1990;157:232-8.<br><br>Cantopher T, Olivieri S, Cleave N, Edwards JG. Chronic benzodiazepine dependence. A comparative study of abrupt withdrawal under propranolol cover versus gradual withdrawal. Br J Psychiatry. 1990;156:406-11.<br><br>Di Costanzo E, Rovea A. [The prophylaxis of benzodiazepine withdrawal syndrome in the elderly: the effectiveness of carbamazepine. Double-blind study vs. placebo]. Minerva Psichiatr. 1992;33(4):301-4.<br><br>Edinger JD, Sampson WS. A primary care "friendly" cognitive behavioral insomnia therapy. Sleep. 2003;26(2):177-82.

		Elsesser K, Sartory G, Maurer J. The efficacy of complaints management training in facilitating benzodiazepine withdrawal. Behav Res Ther. 1996;34(2):149-56.<br><br>Fraser D, Peterkin GS, Gamsu CV, Baldwin PJ. Benzodiazepine withdrawal: a pilot comparison of three methods. Br J Clin Psychol. 1990;29(2):231-3.<br><br>Friedman L, Bliwise DL, Yesavage JA, Salom SR. A preliminary study comparing sleep restriction and relaxation treatments for insomnia in older adults. J Gerontol. 1991;46(1):P1-8.<br><br>Garcia-Borreguero D, Bronisch T, Apelt S, Yassouridis A, Emrich HM. Treatment of benzodiazepine withdrawal symptoms with carbamazepine. Eur Arch Psychiatry Clin Neurosci. 1991;241(3):145-50.<br><br>Garfinkel D, Zisapel N, Wainstein J, Laudon M. Facilitation of benzodiazepine discontinuation by melatonin: a new clinical approach. Arch Intern Med. 1999;159(20):2456-60.<br><br>Gerra G, Marcato A, Caccavari R, Fertonani-Affini G, Fontanesi B, Zaimovic A, et al. Effectiveness of flumazenil (RO 15-1788) in the treatment of benzodiazepine withdrawal. Current Therapeutic Research. 1993;54(5):580-7.<br><br>Higgitt A, Golombok S, Fonagy P, Lader M. Group treatment of benzodiazepine dependence. Br J Addict. 1987;82(5):517-32.<br><br>Lader M, Farr I, Morton S. A comparison of alpidem and placebo in relieving benzodiazepine withdrawal symptoms. Int Clin Psychopharmacol. 1993;8(1):31-6.<br><br>Lader M, Olajide D. A comparison of buspirone and placebo in relieving benzodiazepine withdrawal symptoms. J Clin Psychopharmacol. 1987;7(1):11-5.<br><br>Lichstein KL, Peterson BA, Riedel BW, Means MK, Epperson MT, Aguillard RN. Relaxation to assist sleep medication withdrawal. Behav Modif. 1999;23(3):379-402.<br><br>Morton S, Lader M. Buspirone treatment as an aid to

		benzodiazepine withdrawal. J Psychopharmacol. 1995;9(4):331-5.<br><br>Nathan RG, Robinson D, Cherek DR, Sebastian CS, Hack M, Davison S. Alternative treatments for withdrawing the long-term benzodiazepine user: a pilot study. Int J Addict. 1986;21(2):195-211.<br><br>Onyett SR, Turpin G. Benzodiazepine withdrawal in primary care: a comparison of behavioural group training and individual sessions. Behavioural and Cognitive Psychotherapy. 1988;16(4):297-312.<br><br>Pat-Horenczyk R, Hacohen D, Herer P, Lavie P. The effects of substituting zopiclone in withdrawal from chronic use of benzodiazepine hypnotics. Psychopharmacology (Berl). 1998;140(4):450-7.<br><br>Poyares DR, Guilleminault C, Ohayon MM, Tufik S. Can valerian improve the sleep of insomniacs after benzodiazepine withdrawal? Prog Neuropsychopharmacol Biol Psychiatry. 2002;26(3):539-45.<br><br>Rickels K, Schweizer E, Garcia Espana F, Case G, DeMartinis N, Greenblatt D. Trazodone and valproate in patients discontinuing long-term benzodiazepine therapy: effects on withdrawal symptoms and taper outcome. Psychopharmacology (Berl). 1999;141(1):1-5.<br><br>Riedel B, Lichstein K, Peterson BA, Epperson MT, Means MK, Aguillard RN. A comparison of the efficacy of stimulus control for medicated and nonmedicated insomniacs. Behav Modif. 1998;22(1):3-28.<br><br>Rynn M, Garcia-Espana F, Greenblatt DJ, Mandos LA, Schweizer E, Rickels K. Imipramine and buspirone in patients with panic disorder who are discontinuing long-term benzodiazepine therapy. J Clin Psychopharmacol. 2003;23(5):505-8.<br><br>Sanchez-Craig M, Cappell H, Busto U, Kay G. Cognitive-behavioural treatment for benzodiazepine dependence: a comparison of gradual versus abrupt cessation of drug intake. Br J Addict. 1987;82(12):1317-27.<br><br>Schweizer E, Case WG, Garcia-Espana F, Greenblatt DJ, Rickels K. Progesterone co-administration in patients

		discontinuing long-term benzodiazepine therapy: effects on withdrawal severity and taper outcome. Psychopharmacology (Berl). 1995;117(4):424-9.<br><br>Schweizer E, Rickels K, Case WG, Greenblatt DJ. Carbamazepine treatment in patients discontinuing long-term benzodiazepine therapy. Effects on withdrawal severity and outcome. Arch Gen Psychiatry. 1991;48(5):448-52.<br><br>Schweizer E, Rickels K. Failure of buspirone to manage benzodiazepine withdrawal. Am J Psychiatry. 1986;143(12):1590-2.<br><br>Soeffing JP, Lichstein KL, Nau SD, McCrae CS, Wilson NM, Aguillard RN, et al. Psychological treatment of insomnia in hypnotic-dependant older adults. Sleep Med. 2008;9(2):165-71.<br><br>Taylor DJ, Schmidt-Nowara W, Jessop CA, Ahearn J. Sleep restriction therapy and hypnotic withdrawal versus sleep hygiene education in hypnotic using patients with insomnia. J Clin Sleep Med. 2010;6(2):169-75.<br><br>Tyrer P, Rutherford D, Huggett T. Benzodiazepine withdrawal symptoms and propranolol. Lancet. 1981;1(8219):520-2.<br><br>Vissers FH, Knipschild PG, Crebolder HF. Is melatonin helpful in stopping the long-term use of hypnotics? A discontinuation trial. Pharm World Sci. 2007;29(6):641-6.
No results	4	Baclofen for Improving Benzodiazepine Titration in Benzodiazepine Dependence (BABET). 2023. Available from: https://trialsearch.who.int/Trial2.aspx?TrialID=CTIS2022-502307-30-00.<br><br>Benzodiazepines Deprescribing in Nursing Homes: Intervention Feasibility (END-IT NH). 2023. Available from: https://clinicaltrials.gov/study/NCT05929443.<br><br>Effectiveness of a joint general practitioner-pharmacist intervention on benzodiazepine deprescribing in the elderly (BESTOPH-MG). 2023. Available from: https://clinicaltrials.gov/ct2/show/NCT05765656.<br><br>Wolitzky-Taylor K, Mooney LJ, Otto MW, Metts A, Parsons EM, Hanano M, Ram R. Augmenting the efficacy

		of benzodiazepine taper with telehealth-delivered cognitive behavioral therapy for anxiety disorders in patients using prescription opioids: A pilot randomized controlled trial. Contemp Clin Trials. 2023;133:107334.

Supplement 3: Risk of bias of trials that compared education of patients with usual care

Trial		Randomization	Deviations from the intended intervention	Missing outcome data	Measurement of outcome	Selection of the reported results	Carryover or period effects	Identification or recruitment of participants into clusters
**Discontinuation of BSH**								
Cormack, 1994		Probably high	Probably high	High	Probably low	Probably low		
Heather, 2004		Probably high	Probably high	Low	High	Probably low		
Salonoja, 2010		Probably low	Probably high	Probably low	High	Probably low		
Tannenbaum, 2014		Probably low	Probably low	Probably low	Probably low	Low		Low
Navy, 2018		Probably high	Probably high	Low	Probably high	Probably low		
Kuntz, 2019		Probably high	Probably high	Low	Probably high	Probably low		
Mak, 2022		Probably low	Probably low	Low	Probably high	Low		
Gardner, 2024		Low	Probably high	Probably low	High	Low		
**Dropouts during intervention**								
Heather, 2004		Probably high	Probably high	Low	High	Probably low		
Tannenbaum, 2014		Probably low	Probably low	Low	Probably low	Probably low		Low
Kuntz, 2019		Probably high	Probably high	Low	High	Probably low		
Mak, 2022		Probably low	Probably low	Low	High	Probably low		
**Number of prescriptions**								
Kuntz, 2019		Probably high	Probably high	Low	Probably low	Probably low		
**Physical function**								
Gardner, 2024	SF-12 (physical health component score)	Low	Probably high	Probably low	High	Probably low		
**Mental health**								
Gardner, 2024	SF-12 (mental health component score)	Low	Probably high	Probably low	High	Probably low		
Gardner, 2024	Generalized Anxiety Disorder 7-item scale	Low	Probably high	Probably low	High	Low		
**Signs and symptoms of insomnia**								
Gardner, 2024	Insomnia Severity Index	Low	Probably high	Probably low	High	Low		
**Total sleep time**								
Gardner, 2024	Total sleep time	Low	Probably high	Probably low	High	Low		
**Sleep efficiency**								
Gardner, 2024	Sleep efficiency	Low	Probably high	Probably low	High	Low		
**Sleep onset latency**								
Gardner, 2024	Sleep onset latency	Low	Probably high	Probably low	High	Low		

Low risk of bias
Probably low risk of bias
Probably high risk of bias
High risk of bias

Supplement 4: Risk of bias of trials that compared education of physicians against usual care

Trial	Randomization	Deviations from the intended intervention	Missing outcome data	Measurement of outcome	Selection of the reported results	Carryover or period effects	Identification or recruitment of participants into clusters
	**Discontinuation of BSH**						
Avorn, 2013	Probably high risk of bias	Probably high risk of bias	High risk of bias	Probably low risk of bias	Probably low risk of bias		Low risk of bias

Low risk of bias
Probably low risk of bias
Probably high risk of bias
High risk of bias

Supplement 5: Risk of bias of trials that compared education of patients and physicians against usual care

Trial	Randomization	Deviations from the intended intervention	Missing outcome data	Measurement of outcome	Selection of the reported results	Carryover or period effects	Identification or recruitment of participants into clusters
**Discontinuation of BSH**							
Phelan 2024	Low risk of bias	Probably high risk of bias	Probably low risk of bias	Probably low risk of bias	Probably low risk of bias		Low risk of bias

- Low risk of bias
- Probably low risk of bias
- Probably high risk of bias
- Definitely high risk of bias

Supplement 6: Risk of bias of trials that compared cognitive behavioral therapy (CBT) against usual care

Trial		Randomization	Deviations from the intended intervention	Missing outcome data	Measurement of outcome	Selection of the reported results	Carryover or period effects	Identification or recruitment of participants into clusters
**Discontinuation of BSH**								
Baillargeon, 2003		Probably high risk of bias	Probably high risk of bias	Low risk of bias	Low risk of bias	Probably low risk of bias		
Morgan, 2003, 2004		Probably high risk of bias	Probably high risk of bias	High risk of bias	High risk of bias	Probably low risk of bias	Low risk of bias	Low risk of bias
Morin, 2004		Probably high risk of bias	Probably high risk of bias	Probably high risk of bias	Low risk of bias	Probably low risk of bias		
Voshaar, 2003, 2006		Probably low risk of bias	Probably high risk of bias	High risk of bias	Probably low risk of bias	Probably low risk of bias		
Belleville, 2007		Probably high risk of bias	Probably high risk of bias	High risk of bias	High risk of bias	Probably low risk of bias		
Lichstein, 2013		Probably high risk of bias	Probably high risk of bias	Probably low risk of bias	High risk of bias	Probably low risk of bias		
Sandlund, 2017		Low risk of bias	Probably high risk of bias	High risk of bias	High risk of bias	Probably low risk of bias		
Coteur, 2022		Low risk of bias	Probably high risk of bias	High risk of bias	Low risk of bias	Low risk of bias		Low risk of bias
**Dropouts during intervention**								
Baillargeon, 2003		Probably high risk of bias	Probably high risk of bias	Low risk of bias	High risk of bias	Probably low risk of bias		
Morgan, 2003, 2004		Probably high risk of bias	Probably high risk of bias	Low risk of bias	High risk of bias	Probably low risk of bias	Low risk of bias	Low risk of bias
Morin, 2004		Probably high risk of bias	Probably high risk of bias	Low risk of bias	High risk of bias	Probably low risk of bias		
Belleville, 2007		Probably high risk of bias	Probably high risk of bias	Low risk of bias	High risk of bias	Probably low risk of bias		
Lichstein, 2013		Probably high risk of bias	Probably high risk of bias	Low risk of bias	High risk of bias	Probably low risk of bias		
Sandlund, 2017		Low risk of bias	Probably high risk of bias	Low risk of bias	High risk of bias	Probably low risk of bias		
Ayabe, 2018		Low risk of bias	Probably high risk of bias	Low risk of bias	High risk of bias	Probably low risk of bias		
Coteur, 2022		Low risk of bias	Probably high risk of bias	Low risk of bias	High risk of bias	Probably low risk of bias		Low risk of bias
**Quality of life**								
Voshaar, 2003, 2006	Health Utility Index	Probably low risk of bias	Probably high risk of bias	High risk of bias	High risk of bias	Probably low risk of bias		
**Physical function**								
Morgan, 2003, 2004	SF-36 (physical functioning scale)	Probably high risk of bias	Probably high risk of bias	High risk of bias	High risk of bias	Probably low risk of bias	Low risk of bias	Low risk of bias
Voshaar, 2003, 2006	SF-36 (physical functioning scale)	Probably low risk of bias	Probably high risk of bias	High risk of bias	High risk of bias	Probably low risk of bias		
Belleville, 2007	SF-36 (physical health component score)	Probably high risk of bias	Probably high risk of bias	High risk of bias	High risk of bias	Probably low risk of bias		
**Mental health**								
Morgan, 2003, 2004	SF-36 (mental health scale)	Probably high risk of bias	Probably high risk of bias	High risk of bias	High risk of bias	Probably low risk of bias	Low risk of bias	Low risk of bias
Voshaar, 2003, 2006	SF-36 (mental health scale)	Probably low risk of bias	Probably high risk of bias	High risk of bias	High risk of bias	Probably low risk of bias		
Belleville, 2007	SF-36 (mental health component score)	Probably high risk of bias	Probably high risk of bias	High risk of bias	High risk of bias	Probably low risk of bias		
Belleville, 2007	Beck Depression Inventory	Probably high risk of bias	Probably high risk of bias	High risk of bias	High risk of bias	Probably low risk of bias		
**Cognitive function**								
Voshaar, 2003, 2006	Delayed recall 15-words test	Probably low risk of bias	Probably high risk of bias	High risk of bias	High risk of bias	Probably low risk of bias		
**Signs and symptoms of insomnia**								
Morin, 2004	Insomnia Severity Index	Probably high risk of bias	Probably high risk of bias	Probably high risk of bias	High risk of bias	Probably low risk of bias		
Belleville, 2007	Insomnia Severity Index	Probably high risk of bias	Probably high risk of bias	High risk of bias	High risk of bias	Probably low risk of bias		
Ayabe, 2018	Insomnia Severity Index	Low risk of bias	Probably high risk of bias	High risk of bias	High risk of bias	Low risk of bias		
**Sleep symptoms and sleep efficiency**								
Morgan, 2003, 2004	Pittsburgh Sleep Quality Index	Probably high risk of bias	Probably high risk of bias	High risk of bias	High risk of bias	Probably low risk of bias	Low risk of bias	Low risk of bias
Ayabe, 2018	Pittsburgh Sleep Quality Index	Low risk of bias	Probably high risk of bias	High risk of bias	High risk of bias	Low risk of bias		
**Sleep efficiency**								
Morin, 2004	Sleep efficiency	Probably high risk of bias	Probably high risk of bias	Probably high risk of bias	Low risk of bias	Probably low risk of bias		
Belleville, 2007	Sleep efficiency	Probably high risk of bias	Probably high risk of bias	High risk of bias	High risk of bias	Probably low risk of bias		
**Total sleep time**								
Morgan, 2003, 2004	Total sleep time (hours)	Probably high risk of bias	Probably high risk of bias	High risk of bias	High risk of bias	Probably low risk of bias	Low risk of bias	Low risk of bias
Morin, 2004	Total sleep time (hours)	Probably high risk of bias	Probably high risk of bias	Probably high risk of bias	Low risk of bias	Probably low risk of bias		
Belleville, 2007	Total sleep time (hours)	Probably high risk of bias	Probably high risk of bias	High risk of bias	High risk of bias	Probably low risk of bias		
Lichstein, 2013	Total sleep time (hours)	Probably high risk of bias	Probably high risk of bias	Probably low risk of bias	Low risk of bias	Probably low risk of bias		
**Sleep onset latency**								
Morgan, 2003, 2004	Sleep onset latency (minutes)	Probably high risk of bias	Probably high risk of bias	High risk of bias	High risk of bias	Probably low risk of bias	Low risk of bias	Low risk of bias
Morin, 2004	Sleep onset latency (minutes)	Probably high risk of bias	Probably high risk of bias	Probably high risk of bias	Low risk of bias	Probably low risk of bias		
Lichstein, 2013	Sleep onset latency (minutes)	Probably high risk of bias	Probably high risk of bias	Probably low risk of bias	Low risk of bias	Probably low risk of bias		
**Drug free nights**								
Morgan, 2003, 2004	Drug-free nights	Probably high risk of bias	Probably high risk of bias	High risk of bias	High risk of bias	Probably low risk of bias	Low risk of bias	Low risk of bias

Low risk of bias
Probably low risk of bias
Probably high risk of bias
High risk of bias

Supplement 7: Risk of bias of trials that compared medication review against usual care

Discontinuation of BSH

Trial	Randomization	Deviations from the intended intervention	Missing outcome data	Measurement of outcome	Selection of the reported results	Carryover or period effects	Identification or recruitment of participants into clusters
Strauven, 2019	Probably high risk of bias	Probably high risk of bias	Probably high risk of bias	Probably high risk of bias	Low risk of bias		Low risk of bias
Sibille, 2021, 2023	Probably high risk of bias	Probably high risk of bias	Probably low risk of bias	Definitely high risk of bias	Probably low risk of bias		Low risk of bias

- Low risk of bias
- Probably low risk of bias
- Probably high risk of bias
- Definitely high risk of bias

Supplement 8: Risk of bias of trials that compared mindfulness against usual care

Trial		Randomization	Deviations from the intended intervention	Missing outcome data	Measurement of outcome	Selection of the reported results	Carryover or period effects	Identification or recruitment of participants into clusters
**Dropouts during intervention**								
Barros, 2021		Probably high risk of bias	Probably high risk of bias	Low risk of bias	High risk of bias	Probably low risk of bias		
**Signs and symptoms of insomnia**								
Barros, 2021	Insomnia Severity Index	Probably high risk of bias	Probably high risk of bias	Probably high risk of bias	High risk of bias	Low risk of bias		

Low risk of bias ●
Probably low risk of bias ●
Probably high risk of bias ●
High risk of bias ●

Supplement 9: Risk of bias of trials that addressed pharmacist-led interventions

Trial	Randomization	Deviations from the intended intervention	Missing outcome data	Measurement of outcome	Selection of the reported results	Carryover or period effects	Identification or recruitment of participants into clusters
**<u>Multicomponent Intervention involving pharmacists vs educational Intervention for pharmacists</u>**							
			**Discontinuation of BSH**				
van de Steeg-van Gompel 2009	Probably low risk of bias	Probably high risk of bias	Probably high risk of bias	Probably low risk of bias	Probably low risk of bias		
**<u>Pharmacist-led educational intervention</u>**							
			**Discontinuation of BSH**				
Martin 2018	Probably low risk of bias	Probably high risk of bias	Low risk of bias	Low risk of bias	Low risk of bias		
**<u>Clinincal pharmacy series in nursing homes vs usual care</u>**							
			**Number of prescriptions**				
Roberts, 2001	Probably high risk of bias	Probably high risk of bias	Probably low risk of bias	Probably low risk of bias	Probably low risk of bias		

Low risk of bias
Probably low risk of bias
Probably high risk of bia
High risk of bias

Supplement 10: Risk of bias of trials that addressed pharmacologic-assisted tapering or abrupt withdrawal

Trial		Randomization	Deviations from the intended intervention	Missing outcome data	Measurement of outcome	Selection of the reported results	Carryover or period effects	Identification or recruitment of participants into clusters
**Melatonin-assisted tapering vs tapering alone**								
	**Discontinuation of BSH**							
Lähteenmäki, 2013		Probably low risk of bias	Probably high risk of bias	Probably low risk of bias	Probably low risk of bias	Probably low risk of bias		
	**Dropouts during intervention**							
Lähteenmäki, 2013		Probably low risk of bias	Low risk of bias	Low risk of bias	Low risk of bias	Probably low risk of bias		
**Paroxetine-assisted tapering vs tapering alone**								
	**Discontinuation of BSH**							
Nakao, 2006		Probably high risk of bias	Probably high risk of bias	Probably low risk of bias	Probably high risk of bias	Probably low risk of bias		
	**Dropouts during intervention**							
Nakao, 2006		Probably high risk of bias	Probably high risk of bias	Probably low risk of bias	High risk of bias	Probably low risk of bias		
	**Mental health**							
Nakao, 2006	Hamilton Rating Scale for Anxiety	Probably high risk of bias	Probably high risk of bias	Probably low risk of bias	High risk of bias	Probably low risk of bias		
Nakao, 2006	Hamilton Rating Scale for Depression	Probably high risk of bias	Probably high risk of bias	Probably low risk of bias	High risk of bias	Probably low risk of bias		
**Ramelteon-assisted tapering vs tapering alone**								
	**Discontinuation of BSH**							
Zee 2008		Probably low risk of bias	Low risk of bias	High risk of bias	Low risk of bias	Probably high risk of bias		
	**Dropouts during intervention**							
Zee 2008		Probably low risk of bias	Low risk of bias	Low risk of bias	Low risk of bias	Probably low risk of bias		
**Abrupt withdrawal with propranolol vs abrupt withdrawal alone**								
	**Dropouts during intervention**							
Tyrer, 1981		Probably low risk of bias	Low risk of bias	Probably low risk of bias	Low risk of bias	Probably low risk of bias		
**Dothiepin-assisted tapering vs tapering alone**								
	**Dropouts during intervention**							
Tyrer, 1996		Probably low risk of bias	Low risk of bias	Low risk of bias	Low risk of bias	Probably low risk of bias		
	**Mental health**							
Tyrer, 1996	Hospital Anxiety and Depression Scale- anxiety subscale	Probably low risk of bias	Low risk of bias	High risk of bias	Low risk of bias	Probably low risk of bias		
Tyrer, 1996	Hospital Anxiety and Depression Scale- depression subscale	Probably low risk of bias	Low risk of bias	High risk of bias	Low risk of bias	Probably low risk of bias		
**Abrupt withdrawal with lormetazepam vs abrupt withdrawal alone**								
	**Discontinuation of BSH**							
Petrovic, 2002		Probably low risk of bias	Low risk of bias	Probably low risk of bias	Low risk of bias	Probably low risk of bias		
	**Dropouts during intervention**							
Petrovic, 2002		Probably low risk of bias	Low risk of bias	Low risk of bias	Low risk of bias	Probably low risk of bias		
	**Sleep symptoms and sleep efficiency**							
Petrovic, 2002	Pittsburgh Sleep Quality Index	Probably low risk of bias	Low risk of bias	Probably high risk of bias	Low risk of bias	Probably low risk of bias		
**Abrupt withdrawal with oxazepam, flumazenil vs oxazepam alone**								
	**Discontinuation of BSH**							
Gerra, 2002		Probably high risk of bias	Probably high risk of bias	Probably low risk of bias	Low risk of bias	Probably low risk of bias		
**Tapering of diazepam vs lorazepam vs bromazepam**								
	**Discontinuation of BSH**							
Murphy, 1991		Probably low risk of bias	Low risk of bias	Probably low risk of bias	Low risk of bias	Probably low risk of bias		
	**Dropouts during intervention**							
Murphy, 1991		Probably low risk of bias	Low risk of bias	Low risk of bias	Low risk of bias	Probably low risk of bias		
	**Mental health**							
Murphy, 1991	Comprehensive Psychopathological Rating Scale	Probably low risk of bias	Low risk of bias	High risk of bias	Low risk of bias	Probably low risk of bias		

Low risk of bias
Probably low risk of bias
Probably high risk of bias
High risk of bias

Supplement 11: Table of trial characteristics for trials that addressed tapering

Trial	Registration	Funding	Design	Country	Male (%)	Mean age	Mean duration of benzodiazepine use/sedative hypnotics (years)	Using benzodiazepines/ sedative hypnotics for insomnia (%)	Using benzodiazepines/ sedative hypnotics for anxiety (%)	Using benzodiazepines/ sedative hypnotics for conditions other than insomnia (%)	Interventions	Number of patients	Description
**Habraken, 1997**	NR	Government	Parallel RCT	Belgium	18.18	84	≥1	NR	NR	NR	taper	27	Tapering (25% reduction at 1-week interval for 3 weeks, then 12.5% reduction at 1-interval for 2 weeks)
											usual care	28	Usual care
**Voshaar, 2003, 2006**	NR	Government	Parallel RCT	Netherlands	70	63.1	13.5	NR	NR	NR	taper	73	Tapering (25% reduction of dosage per week with the option to divide the final step to 12.5% over 4 days)
											usual care	34	Usual care
**Nakao, 2006**	NR	NR	Parallel RCT	Japan	33.33	58.5	6.03	NR	NR	NR	taper	23	Tapering (25% reduction of dosage at 2-week intervals)
											usual care	21	Usual care
**Vicens, 2014, 2018 (BENZORED)**	ISRCTN13024375	Government	Cluster RCT	Spain	28.02	64	4 (usual care), 5 (taper without follow-up), 5 (taper with follow-up)*	68.71	65.06	29.54 (depression), 11.18 (pain)	taper (with follow-up)	191	Educational interview of patients by GPs to provide information on benzodiazepines, the risks of long term use, reassurance about reducing medication; Patients received a self-help leaflet on improving sleep; Tapering (10-25% reduction at 2-3 week intervals); Follow-up appointments with their GP every 2–3 weeks until the end of the dose reduction during which GPs reinforced education, reassured patients regarding withdrawal symptoms, and obtained patient agreement for the next step in dose reduction
											taper (without follow-up)	168	Educational interview of patients by GPs to provide information on benzodiazepines, the risks of long term use, reassurance about reducing medication; Patients received a self-help leaflet on improving sleep; Tapering (10-25% reduction at 2–3-week intervals); Written instructions reinforcing educational information
											usual care	173	Usual care

**Kosto, 2023**	NR	None	Parallel RCT	Israel	49.5	75.49	≥0.25	100	NR	NR	taper	55	Patients received written materials for improving sleep hygiene, discontinuing benzodiazepines and z-drugs, and a tapering reduction schedule over 7 weeks
											usual care	46	Usual care
**Fung, 2024 (SWITCH)**	NCT03687086	Industry, Government, Institutional	Parallel RCT	United States of America	65.42	69.2	≥0.25	100	NR	NR	Taper (masked), CBT	92	Patients received eight individual 60 minute CBT, research psychologist-led, in-person/video sessions plus up to two 30 minute drug withdrawal symptom check-in sessions over 9 weeks. CBT consisted of stimulus control, sleep restriction, cognitive therapy, relaxation techniques, and a weekly standard sleep diary. Masked tapering (25% reduction of dosage per week over 9 weeks), involved patients receiving 1 benzodiazepine capsule QD, containing progressively larger amounts of inert filler.
											Taper (unmasked), CBT	96	Patients received eight individual 60 minute CBT, research psychologist-led, in-person/video sessions plus up to two 30 minute drug withdrawal symptom check-in sessions over 9 weeks. CBT consisted of stimulus control, sleep restriction, cognitive therapy, relaxation techniques, and a weekly standard sleep diary. Tapering (25% reduction of dosage per week over 9 weeks), involved patients using a paper schedule and a pill cutter.

NR=Not reported
*Median

Supplement 12: Summary of findings for the comparison of tapering against usual care

Outcome	Study results and measurements	Absolute effect estimates<br>Usual Care / Taper	Certainty of the evidence (Quality of evidence)	Summary
Discontinuation	Odds ratio: 1.72 (CI 95% 1.23 - 2.41) Based on data from 735 participants in 3 studies	Usual Care: **130** per 1000; Taper: **204** per 1000<br>Difference: **74 more per 1000** (CI 95% 25 more - 135 more)	**Low**<br>Due to serious risk of bias, Due to serious imprecision	Tapering may have little or no effect on the proportion of patients who discontinue benzodiazepines/sedative hypnotics compared to usual care.
Dropouts During Intervention	Odds ratio: 1.28 (CI 95% 0.36 - 4.56) Based on data from 506 participants in 2 studies	Usual Care: **110** per 1000; Taper: **137** per 1000<br>Difference: **27 more per 1000** (CI 95% 67 fewer - 250 more)	**Low**<br>Due to serious imprecision, Due to serious risk of bias	Tapering may have little or no effect on dropouts during intervention compared to usual care.
Quality of Life (Health Utility Index)	Scale: 0 - 1 High better Based on data from 72 participants in 1 study	Difference: **MD 0.11 lower** (CI 95% 0.25 lower - 0.03 higher)	**Low**<br>Due to serious risk of bias, Due to serious imprecision	Tapering may worsen quality of life compared to usual care.
Function (SF-36 Physical Functioning Scale)	Scale: 0 - 100 High better Based on data from 137 participants in 1 study	Difference: **MD 7 lower** (CI 95% 19 lower - 5 higher)	**Low**<br>Due to serious risk of bias, Due to serious imprecision	Tapering may worsen physical function compared to usual care.
Mental Health (SF-36 Mental Health Scale)	Scale: 0 - 100 High better Based on data from 85 participants in 1 study	Difference: **MD 5.00 lower** (CI 95% 19.94 lower - 9.94 higher)	**Low**<br>Due to serious risk of bias, Due to serious imprecision	Tapering may worsen mental health compared to usual care.
Mental Health (HADS-A)	Scale: 0 - 21 Lower better Based on data from 506 participants in 1 study	Difference: **MD 0 higher** (CI 95% 0.87 lower - 0.87 higher)	**Moderate**<br>Due to serious risk of bias	Tapering probably has little or no effect on mental health (anxiety) compared to usual care.
Mental Health (HADS-D)	Scale: 0 - 21 Lower better Based on data from 506 participants in 1 study	Difference: **MD 0 higher** (CI 95% 0.62 lower - 0.62 higher)	**Moderate**<br>Due to serious risk of bias	Tapering probably has little or no effect on mental health (depression) compared to usual care.
Mental Health (Hamilton Rating Scale for Anxiety)	Scale: 0 - 56 Lower better Based on data from 44 participants in 1 study	Difference: **MD 5.40 higher** (CI 95% 1.67 higher - 9.13 higher)	**Low**<br>Due to serious risk of bias, Due to serious imprecision	Tapering may worsen mental health (anxiety) compared to usual care.
Mental Health (Hamilton Rating Scale for Depression)	Scale: 0 - 52 Lower better Based on data from 44 participants in 1 study	Difference: **MD 0.10 higher** (CI 95% 1.95 lower - 2.15 higher)	**Moderate**<br>Due to serious risk of bias	Tapering probably has little or no effect on mental health (depression) compared to usual care.
Cognitive Function (Delayed Recall 15-words Test)	Scale: 0 - 15 High better Based on data from 107 participants in 1 study	Difference: **MD 0.40 lower** (CI 95% 1.47 lower - 0.67 higher)	**Low**<br>Due to serious risk of bias, Due to serious imprecision	Tapering may have little or no effect on cognitive function compared to usual care.

Supplement 13: Table of trial characteristics for trials that investigated education of patients

Trial	Registration	Funding	Design	Country	Male (%)	Mean age	Mean duration of benzodiazepine use/sedative hypnotics (years)	Using benzodiazepines /sedative hypnotics for insomnia (%)	Using benzodiazepines/ sedative hypnotics for anxiety (%)	Using benzodiazepines/ sedative hypnotics for conditions other than insomnia (%)	Interventions	Number of patients	Description
**Tinetti, 1994**	NR	Government	Parallel RCT	United States of America	30.9	77.91	NR	NR	NR	NR	education (patients)	153	Physical therapist visited patients' homes to assess risk factors for falls, including benzodiazepine use; Education of patients about benzodiazepine and sedative hypnotic tapering and withdrawal and alternative options like sleep restriction
											usual care	148	Usual care
**Cormack, 1994**	NR	Government, Non-profit	Parallel RCT	United Kingdom	20.57	69	15*	NR	NR	NR	education (patients) with letter	65	Patients received a letter from their GP asking them to reduce or stop benzodiazepine medication gradually
											education (patients) with letter and additional educational materials	75	Patients received a letter from their GP to reduce or stop benzodiazepine medication gradually; Patients received educational materials every month for four months with advice about reducing medication, including practical suggestions for coping without drugs
											usual care	69	Usual care

**Heather, 2004**	NR	Government	Parallel RCT	United Kingdom	23	69.16	14.08	NR	NR	NR	education (patients) with letter, self-help booklet, and invitation to see physician	98	Patients received a letter inviting them to see their GP for a medication review that included information about benzodiazepines, beneficial reasons of reducing medication, and a timetable that could be used to plan withdrawal; Patients received a self-help booklet and leaflet about hypnotic medications and sleeping problems; Researchers met GPs and provided guidance on how the consultation should be performed
											education (patients) with letter	93	Patients received a letter from their GP asking patients to reduce or stop benzodiazepine medication gradually
											usual care	93	Usual care
**Tenwolde, 2008**	NR	Government	Parallel RCT	Netherlands	31.9	62.3	8.1	53.1	25.1	NR	education (patients) with three tailored letters	310	Patients received three letters, each 3 pages, at 1-month intervals that were designed to increase positive outcome expectations of discontinuing benzodiazepine use, reduce the positive outcome expectations of benzodiazepine use, and increase self-efficacy expectations by offering skills to reach abstinence; Letters were tailored, including personalization, feedback, and adaptation
											education (patients) with one tailored letter	278	Patients received a single letter 5 to 6 pages long that was designed to increase positive outcome expectations of discontinuing benzodiazepine use, reduce the positive outcome

													expectations of benzodiazepine use, and increase self-efficacy expectations by offering skills to reach abstinence; Letters were tailored, including personalization, feedback, and adaptation
											education (patients) with generic letter	273	Patients received a letter describing disadvantages of benzodiazepines and providing brief advice on discontinuation
**Salonoja, 2010**	NCT00247546	Government, Institutional, Non-profit	Parallel RCT	Finland	16.1	72.85	NR	NR	NR	NR	education (patients)	259	One time counselling by geriatricians for patients to taper inappropriate drugs that could cause falls; Written instructions to patients to facilitate stepwise reduction of drugs; Included notes in medical record for GPs; Patients attended a 1-hour lecture on adverse effects of psychotropics
											usual care	269	Usual care
**Tannenbaum, 2014 (EMPOWER)**	NCT01148186	Government	Cluster RCT	Canada	30.69	74.8	10.42	60.39	47.49	2.96 (pain), 6.65 (other)	education (patients)	148	Patients received a mailed educational booklet that included self-assessment component about the risks of benzodiazepine use, harms, education about drug interactions, peer champion stories to augment self-efficacy, substitutes for insomnia and/or anxiety and visual tapering recommendations
											usual care	155	Usual care
**Navy, 2018**	NR	Institutional	Parallel RCT	United States of America	35.99	73.4	≥0.25	NR	NR	NR	education (patients)	173	Patients received a mailed letter from pharmacist outlining drug risks, side effects, organizations that recommend against the drug, alternate treatments, and the option to discuss other options with pharmacist; Mailed letter was developed with input from geriatric clinical pharmacist and medical

													director of geriatrics and medicare; Pharmacists provided alternative options like melatonin, trazodone, mirtazapine, and CBT; Optional individualized taper plan developed by clinical pharmacist and patient's physician; Pharmacists monitored patients for withdrawal symptoms via telephone
											usual care	173	Usual care
**Kuntz, 2019**	NR	Government	Parallel RCT	United States of America	33.56	70	NR	NR	NR	NR	education (patients)	50	Patients received a mailed letter from physician encouraging patients to reconsider Z-drugs, an educational brochure about drug-induced harms, suggestions for alternatives to treat insomnia, a visual tapering schedule, and a quiz about Z-drug risks and alternatives
											education (patients) with pharmacist follow-up	49	Patients received a mailed letter from physician encouraging patients to reconsider Z-drugs, an educational brochure about drug-induced harms, suggestions for alternatives to treat insomnia, a visual tapering schedule, and a quiz about Z-drug risks and alternatives; Patients received a call from pharmacist to discuss and reinforce information in the educational mailing, assess patient barriers to drug discontinuation, and provide personalized guidance on tapering, and answer questions
											usual care	50	Usual care
**Mak, 2022**	ISRCTN11530968	Government, Non-profit	Parallel RCT	United States of America	94	71.9	≥0.25	74	NR	NR	education (patients) with mailed brochure	946	Patients received a mailed brochure that included self-assessment component about the risks of benzodiazepine use, harms,

													education about drug interactions, peer champion stories to augment self-efficacy, substitutes for insomnia and/or anxiety, and a link to a CBT website
											education (patients) with mailed brochure and telephone reinforcement by researcher	120	Patients received a mailed brochure that included self-assessment component about the risks of benzodiazepine use, harms, education about drug interactions, peer champion stories to augment self-efficacy, substitutes for insomnia and/or anxiety, and a link to a CBT website; Patients received a telephone reinforcement call by a researcher within 1-2 months of randomization
											usual care	943	Patients received a mailed brochure including general information about the importance of sleep
**Gardner, 2024 (YAWNS NB)**	NCT04406103	Government	Parallel RCT	Canada	35.93	72.1	11.4	87.08	12.04	NR	YAWNS-1	195	Patients received educational materials consisting of a cover letter, 2 Sleepwell booklets (“How to Stop Sleeping Pills” and “How to Get Your Sleep Back”), and website resource
											YAWNS-2	193	Patients received educational materials consisting of 2 EMPOWER booklets ("You may be at risk" AND "How to get a good night's sleep without sleeping pills")
											usual care	192	Usual care

NR=Not reported
*Median

Supplement 14: Summary of findings for the comparison of education of patients against usual care

Outcome	Study results and measurements	Absolute effect estimates		Certainty of the evidence (Quality of evidence)	Summary
		Usual Care	Education (Patients)		
Discontinuation	Odds ratio: 2.53 (CI 95% 1.58 - 4.04) Based on data from 4055 participants in 8 studies	**130** per 1000	**274** per 1000	**Low** Due to serious risk of bias, Due to serious imprecision	Education (patients) may increase the proportion of patients who discontinue benzodiazepines/sedative hypnotics compared to usual care.
		Difference: **144 more per 1000** (CI 95% 61 more - 246 more)			
Dropouts During Intervention	Odds ratio: 1.41 (CI 95% 0.76 - 2.63) Based on data from 587 participants in 2 studies	**110** per 1000	**148** per 1000	**Low** Due to serious risk of bias, Due to serious imprecision	Education (patients) may have little or no effect on dropouts during intervention compared to usual care.
		Difference: **38 more per 1000** (CI 95% 24 fewer - 135 more)			
Function (SF-12 Physical Component Score)	Scale: 0 - 100 High better Based on data from 521 participants in 1 study	Difference: **MD 0.1 higher** (CI 95% 1.42 lower - 1.62 lower)		**Moderate** Due to serious risk of bias	Education (patients) probably has little or no effect on physical health compared to usual care.
Mental health (SF-12 Mental Component Score)	Scale: 0 - 100 High better Based on data from 521 participants in 1 study	Difference: **MD 1.56 higher** (CI 95% 0.12 lower - 3.23 higher)		**Moderate** Due to serious risk of bias	Education (patients) probably has little or no effect on mental health compared to usual care.
Mental health (Generalized Anxiety Disorder 7-item scale)	Scale: 0 - 21 Lower better Based on data from 521 participants in 1 study	Difference: **MD 1.10 lower** (CI 95% 1.97 lower - 0.23 lower)		**Moderate** Due to serious risk of bias	Education (patients) probably has little or no effect on mental health (anxiety) compared to usual care.
Signs and Symptoms of Insomnia (Insomnia Severity Index)	Scale: 0 - 21 Lower better Based on data from 521 participants in 1 study	Difference: **MD 1.9 lower** (CI 95% 2.97 lower - 0.83 lower)		**Moderate** Due to serious risk of bias	Education (patients) probably has little or no effect on signs and symptoms of insomnia compared to usual care.
Total Sleep Time (Hours)	Scale: High better Based on data from 521 participants in 1 study	Difference: **MD 0.06 higher** (CI 95% 0.25 lower - 0.37 higher)		**Moderate** Due to serious risk of bias	Education (patients) probably has little or no effect on total sleep time compared to usual care.
Sleep Onset Latency (Minutes)	Scale: Lower better Based on data from 521 participants in 1 study	Difference: **MD 13.34 lower** (CI 95% 23.19 lower - 3.49 lower)		**Moderate** Due to serious risk of bias	Education (patients) probably has little or no effect on sleep onset latency compared to usual care.
Sleep Efficiency	Scale: 0 - 100 High better Based on data from 521 participants in 1 study	Difference: **MD 0.77 higher** (CI 95% 2.34 lower - 3.87 higher)		**Moderate** Due to serious risk of bias	Education (patients) probably has little or no effect on sleep efficiency compared to usual care.
Number of Prescriptions 6 months	Scale: Lower better Based on data from 149 participants in 1 study	**1.9** Mean	**1.0** Mean	**Low** Due to serious risk of bias, Due to serious imprecision	Education (patients) may have little or no effect on the number of prescriptions compared to usual care.
		Difference: **MD 0.90 lower** (CI 95% 1.44 lower - 0.36 lower)			

Supplement 15: Table of trial characteristics for trials that investigated education of physicians

Trial	Registration	Funding	Design	Country	Male (%)	Mean age	Mean duration of benzodiazepine use/sedative hypnotics (years)	Using benzodiazepines/ sedative hypnotics for insomnia (%)	Using benzodiazepines/ sedative hypnotics for anxiety (%)	Using benzodiazepines/ sedative hypnotics for conditions other than insomnia (%)	Interventions	Number of patients	Description
**Avorn, 1992**	NR	Non-profit	Cluster RCT	United States of America	NR	NR	NR	NR	NR	NR	education (physicians)	431	Comprehensive educational program for physicians and nurses in nursing homes; Design of the program was informed by interviews with nurses, nursing assistants, and physicians to identify factors related to prescribing psychoactive drugs and literature reviews addressing management of insomnia, confusion, and agitation in nursing homes; Physicians received visually engaging educational materials and three visits by pharmacist; Nurses and nursing assistants received four training sessions
											usual care	392	Usual care
**deBurgh, 1995**	NR	Government	Cluster RCT	Australia	NR	NR	NR	NR	NR	NR	education (physicians)	NR	Twenty-minute educational visit by medical staff or pharmacist to physicians encouraging counseling of patients; Educational materials including management guidelines, indications for benzodiazepine use, and content for patients on improving sleep; Audio tapes and video series on sleep and relaxation for patients; Follow-up phone call to physician
											usual care	NR	Usual care
**Pimlott, 2003**	NR	Institutional, Non-profit	Cluster RCT	Canada	34.76	76.25	NR	NR	NR	NR	A&F, education (physicians)	168	Physicians received mailed feedback on benzodiazepine prescribing practices compared to peers every 2 months for 6 months; Physicians received evidence-based educational bulletins and patient information sheets

													informed by focus groups with primary care physicians about challenges of safe and effective prescribing of benzodiazepines for elderly patients
											usual care	206	Physicians received mailed educational material and feedback about antihypertensive prescribing for elderly patients
**Midlöv, 2005**	NR	Industry, Government, Institutional	Cluster RCT	Sweden	NR	NR	NR	NR	NR	NR	education (physicians)	NR	Physicians attended two educational in person meetings led by trial physician and pharmacist. The first meeting covered different causes of confusion in the elderly such as medications and reasons for illnesses or infections. The second meeting covered the effects of benzodiazepines and psychotropic drugs in the elderly
											usual care	NR	Usual care
**Pit, 2007**	12605000264684	Government, Institutional	Cluster RCT	Australia	40.52	65+	NR	NR	NR	NR	A&F, education (physicians), financial incentives	539	Physicians received 2 educational sessions by pharmacists about performing medication reviews, information sheets, and were directed to sources of information on prescribing; Physicians received feedback on the number of targeted drugs used by their patients; Physicians were reimbursed for 10 medication reviews and for time with pharmacists; Patients completed Medication Risk Assessment Checklist and physician decided whether to perform Medication Review Checklist for at-risk patients
											usual care	555	Usual care
**Ashworth, 2021**	NR	None	Cluster RCT	Canada	NR	65+	NR	NR	NR	NR	A&F, education (physicians)	NR	Physicians received a prescription profile describing patients to whom the physician may have prescribed harmful doses of opioids and/or benzodiazepines and

		indicates how the physicians prescribing compares to peers; Physicians received a letter from the medical regulatory authority body describing best practices and advice on how to reduce medications in patients receiving higher doses of benzodiazepines and links to external prescribing resources
A&F, education (physicians)	NR	Prescription profile describing potentially harmful prescription of benzodiazepines; Physicians received a letter from the medical regulatory authority body describing best practices and advice on how to reduce medications in patients receiving higher doses of benzodiazepines and links to external prescribing resources; Physicians received a phone call from a pharmacist describing best practices and advice on how to reduce medications in patients receiving higher doses of benzodiazepines and links to external prescribing resources
A&F, education (physicians)	NR	Prescription profile describing potentially harmful prescription of benzodiazepines; Physicians received a letter from the medical regulatory authority body describing best practices and advice on how to reduce medications in patients receiving higher doses of benzodiazepines and links to external prescribing resources; Physicians received a phone call from another physician describing best practices and advice on how to reduce medications in patients receiving higher doses of benzodiazepines and links to external prescribing resources
A&F	NR	Physicians received a

													prescription profile describing patients to whom the physician may have prescribed harmful doses of opioids and/or benzodiazepines and indicates how the physicians prescribing compares to peers
**Vicens, 2022 (BENZORED hybrid type 1)**	ISRCTN28272199	Government, Non-profit	Cluster RCT	Spain	NR	65+	NR	NR	NR	NR	A&F, education (physicians)	NR	Physicians received a 2-hour educational face-to-face workshop by researchers on appropriate procedures to prescribe benzodiazepines and were asked to discuss with their patients harms and benefits of taking benzodiazepines before the first prescription; Physicians received automated monthly feedback; Physicians received access to a webpage that provided information to reinforce the messages of the workshop
											usual care	NR	Usual care

NR=Not reported
*Median

Supplement 16: Summary of findings for the comparison of education of physicians against usual care

Outcome	Study results and measurements	Absolute effect estimates		Certainty of the evidence (Quality of evidence)	Summary
		Usual Care	Education (Patients)		
Discontinuation	Odds ratio: 18.78 (CI 95% 2.31 - 152.69) Based on data from 109 participants in 1 study	**130** per 1000	**737** per 1000	**Very low** Due to serious risk of bias, Due to very serious imprecision	We are uncertain of the effects of education (physicians) on the proportion of patients who discontinue benzodiazepines/sedative hypnotics.
		Difference: **607 more per 1000** (CI 95% 127 more - 828 more)			

Supplement 17: Table of trial characteristics for trials that investigated education of patients and physicians

Trial	Registration	Funding	Design	Country	Male (%)	Mean age	Mean duration of benzodiazepine use/sedative hypnotics (years)	Using benzodiazepines/ sedative hypnotics for insomnia (%)	Using benzodiazepines/ sedative hypnotics for anxiety (%)	Using benzodiazepines/ sedative hypnotics for conditions other than insomnia (%)	Interventions	Number of patients	Description
**Phelan, 2024 (STOP-FALLS)**	NCT05689554	Government	Cluster RCT	United States of America	37.14 (mix of users and non-users)	70.6 (mix of users and non-users)	NR	NR	NR	NR	education (patients, physicians)	1106	Patients received educational materials: medication-specific brochures; self-care handouts with tips for managing symptoms using nonpharmacologic approaches; and a public-domain brochure on ways to prevent falls. Physicians received evidence-based pharmaceutical opinions (EBPOs) and deprescribing pearls, addressing medication risks and alternatives, and tips on how to conduct conversation with patients
											usual care	1261	Usual care

NR=Not reported
*Median

Supplement 18: Summary of findings for the comparison of education of patients and physicians against usual care

Outcome	Study results and measurements	Absolute effect estimates		Certainty of the evidence (Quality of evidence)	Summary
		Usual Care	Education (patients, physicians)		
Discontinuation	Relative risk: 0.97 (CI 95% 0.8 - 1.18) Based on data from 465 participants in 1 study	**130** per 1000	**126** per 1000	**Moderate** Due to serious risk of bias	Education (patients, physicians) probably has little or no effect on the proportion of patients who discontinue benzodiazepines/sedative hypnotics compared to usual care.
		Difference: **4 fewer per 1000** (CI 95% 26 fewer - 23 more)			

Supplement 19: Table of trial characteristics for trials that investigated cognitive behavioral therapy

Trial	Registration	Funding	Design	Country	Male (%)	Mean age	Mean duration of benzodiazepine use/sedative hypnotics (years)	Using benzodiazepines/ sedative hypnotics for insomnia (%)	Using benzodiazepines/ sedative hypnotics for anxiety (%)	Using benzodiazepines/ sedative hypnotics for conditions other than insomnia (%)	Interventions	Number of patients	Description
**Baillargeon, 2003**	NR	Government	Parallel RCT	Canada	42	67.4	12.66	100	NR	0	CBT, taper	35	Tapering (25% reduction of dosage at 1- or 2-week intervals); Cognitive behavioral therapy in groups of five to seven patients by psychologist weekly over 8 weeks, including education about stimulus control and procedures for sleep restrictions, sleep hygiene, and irrational thinking that could exacerbate sleep disorder
											taper	30	Tapering (25% reduction of dosage at 1- or 2-week intervals)
**Morgan, 2003**	NR	Government	Crossover by cluster	United Kingdom	32.54	65.43	13.37	100	0	0	CBT	108	Six 50-minute weekly insomnia CBT treatment sessions by a counsellor that included sleep hygiene, stimulus control procedures, relaxation procedures, and cognitive therapy; Patients were offered a tapering program overseen by a physician; Patients received handouts summarizing therapeutic points and an audiotape of relaxation instructions
											usual care	101	Patients were offered a tapering program overseen by a physician
**Morin, 2004**	NR	Government	Parallel RCT	Canada	50	62.5	19.3	100	0	0	taper	25	Tapering (25% reduction of dosage at 2-week intervals, full suppression by week 10)
											CBT	24	Ten sessions of CBT weekly for 90-minute sessions in small groups of four to six patients led by a psychologist with the following modules: sleep restriction, stimulus control, altering faulty beliefs that exacerbate insomnia, and an education component
											CBT, taper	27	Tapering (25% reduction of dosage at 2-week intervals, full suppression by week 10); Ten sessions of CBT weekly for 90-minute sessions in small groups of

													four to six patients led by a psychologist with the following modules: sleep restriction, stimulus control, altering faulty beliefs that exacerbate insomnia, and an education component
**Voshaar, 2003, 2006**	NR	Government	Parallel RCT	Netherlands	70	63.1	13.5	NR	NR	NR	taper	73	Tapering (25% reduction of dosage per week with the option to divide the final step to 12.5% over 4 days)
											CBT, taper	73	Tapering (25% reduction of dosage per week with the option to divide the final step to 12.5% over 4 days); Five weekly 2-hour group CBT sessions led by psychologists trained in CBT with the following modules: psychoeducation about advantages and disadvantages of benzodiazepines, relaxation exercises, and cognitive restructuring of the interpretation of withdrawal symptoms
											usual care	34	Usual care
**Belleville, 2007**	NR	NR	Parallel RCT	Canada	35.85	55.3	14.1	100	NR	0	taper	25	Tapering (25% reduction at 2-week intervals or at patient's readiness to discontinue) with progressive introduction of drug-free nights; Patients received a brochure describing hypnotic medications and withdrawal procedures; Patients received weekly telephone follow-ups with psychology graduate students to monitor withdrawal effects
											CBT, taper	28	Patients received five self-help CBT booklets that included education on self-management and insomnia, stimulus control, cognitive therapy for changing dysfunctional beliefs, sleep hygiene, and self-evaluation; Tapering (25% reduction at 2 week intervals or at patient's readiness to discontinue) with progressive introduction of drug-free nights; Written brochure describing hypnotic medications and withdrawal procedures; Patients received weekly telephone follow-ups with psychology graduate students to monitor withdrawal effects

**Lichstein, 2013**	NR	Government	Parallel RCT	United States of America	28.57	63.51	4.28	100	0	0	CBT, taper	24	Tapering (gradually reducing an individual's nightly sleep medication to a lowest recommended dose of 1, and gradually eliminating nightly doses) with written instructions; Eight weekly individual sessions of CBT by graduate students that included relaxation, stimulus control instructions, sleep hygiene instructions, and disabusing participants of erroneous, exaggerated, and self-defeating cognition
											placebo biofeedback	23	Tapering (gradually reducing an individual's nightly sleep medication to a lowest recommended dose, and gradually eliminating nightly doses) with written instructions; placebo biofeedback
											taper	23	Tapering with written instructions (gradually reducing an individual's nightly sleep medication to lowest recommended dose of 1, and gradually eliminating nightly doses)
**Bergdahl, 2017**	NCT01765959	Government, Non-profit	Parallel RCT	Sweden	14.04	61	7.3	100	NR	0	CBT (combined)	35	Patients were asked to discontinue hypnotics 3 to 5 days before intervention; Patients received six weekly sessions of group CBT by registered psychologists trained in CBT including education on sleep physiology, coping with sleep problems, sleep restrictions, stimulus restriction, and relaxation techniques
											auricular acupuncture	32	Patients were asked to discontinue hypnotics 3 to 5 days before intervention; Eight sessions (twice per week for 4 weeks) of group Articular Acupuncture according to the National Acupuncture Detoxification Association (NADA) protocol by trained members of the medical staff
**Sandlund 2017 (Sleep Prime)**	NCT01731223	Government	Parallel RCT	Sweden	27.27	54.4	NR	100	NR	NR	CBT, taper	90	Patients received seven 2-hour group CBT nurse-led sessions over 10 weeks, consisting of identifying and changing current maladaptive behaviors and unhelpful thoughts

													that could perpetuate insomnia
											taper	75	Usual care
**Ayabe, 2018**	UMIN000014297	Government, Institutional	Parallel RCT	Japan	41.1	59.8	7.8	100	0	0	CBT, taper	24	Tapering (25% reduction at 2-week interval); Patients attended 5 individual CBT sessions every other week with the following modules: insomnia and sleep hygiene, progressive muscle relaxation, sleep scheduling, and dose reduction of hypnotics
											taper	27	Tapering (25% reduction at 2-week interval); Patients received a sleep hygiene leaflet that included information on restricting caffeine intake, exercising during the day, restricting alcohol intake, and taking a bath ≥ 2 hours before bedtime
**Edinger, 2019**	NCT02831894	Government	Parallel RCT	United States of America	34.6	55.14	9.74	100	0	0	CBT	26	Individual CBT sessions by a psychologist for 4 sessions over 6 weeks with the following modules: discussion of normal sleep, use of constructive worry exercises to address sleep-disruptive worrying, cognitive restructuring to address unhelpful beliefs about sleep, review of all strategies
											CBT, taper (10% reduction)	26	Tapering (10% reduction of dosage at 2-week intervals); Individual CBT sessions by a psychologist for 4 sessions over 6 weeks with the following modules: discussion of normal sleep, use of constructive worry exercises to address sleep-disruptive worrying, cognitive restructuring to address unhelpful beliefs about sleep, review of all strategies
											CBT, taper (25% reduction)	26	Tapering (25% reduction of dosage at 2-week intervals); Individual CBT sessions by a psychologist for 4 sessions over 6 weeks with the following modules: discussion of normal sleep, use of constructive worry exercises to address sleep-disruptive worrying, cognitive restructuring to address unhelpful beliefs about sleep, review of all strategies

**Coteur, 2022 (Big Bird)**	NCT03937180	Institutional	Cluster RCT	Belgium	28.28	61	≥0.5	100	NR	NR	CBT	456	Patients received an interactive E-learning program including education about types of hypnotics, associated effects and risks of hypnotic use, sleep and sleep hygiene, non-pharmacological alternatives, tapering of hypnotics based on the principles of CBT; Patients had the option to add a personal tapering schedule
											usual care	460	Usual care

NR=Not reported
*Median

Supplement 20: Summary of findings for the comparison of CBT against usual care

Outcome	Study results and measurements	Absolute effect estimates: Usual Care	Absolute effect estimates: Cognitive Behavioral Therapy (CBT)	Certainty of the evidence (Quality of evidence)	Summary
Discontinuation	Odds ratio: 1.68 (CI 95% 0.82 - 3.45) Based on data from 1472 participants in 8 studies	**130** per 1000	**201** per 1000	**Very low** Due to serious risk of bias, Due to serious imprecision, Due to serious inconsistency	We are uncertain of the effects of CBT on the proportion of patients who discontinue benzodiazepines/sedative hypnotics compared to usual care.
		Difference: **71 more per 1000** (CI 95% 21 fewer - 210 more)			
Dropouts During Intervention	Odds ratio: 1.24 (CI 95% 0.68 - 2.24) Based on data from 1582 participants in 8 study	**110** per 1000	**133** per 1000	**Low** Due to serious risk of bias, Due to serious imprecision	CBT may have little or no effect on dropouts during intervention compared to usual care.
		Difference: **23 more per 1000** (CI 95% 32 fewer - 107 more)			
Quality of Life (Health Utility Index)	Scale: 0 - 1 High better Based on data from 121 participants in 1 study	Difference: **MD 0.04 higher** (CI 95% 0.05 lower - 0.14 higher)		**Low** Due to serious risk of bias, Due to serious imprecision	CBT may improve quality of life compared to usual care.
Function (SF-36-Physical Health Component Score)	Scale: 0 - 100 High better Based on data from 43 participants in 1 study	Difference: **MD 8.32 lower** (CI 95% 19.60 lower - 2.96 higher)		**Low** Due to serious risk of bias, Due to serious imprecision	CBT may worsen physical function compared to usual care.
Function (SF-36-Physical Functioning Scale)	Scale: 0 - 100 High better Based on data from 268 participants in 2 studies	Difference: **MD 4.40 higher** (CI 95% 1.77 lower - 10.57 higher)		**Low** Due to serious risk of bias, Due to serious imprecision	CBT may have little or no effect on physical function compared to usual care.
Mental Health (SF-36-Mental Health Component Score)	Scale: 0 - 100 High better Based on data from 43 participants in 1 study	Difference: **MD 1.09 lower** (CI 95% 10.90 lower - 8.72 higher)		**Low** Due to serious risk of bias, Due to serious imprecision	CBT may have little or no effect on mental health compared to usual care.
Mental Health (SF-36 Mental Health Scale)	Scale: 0 - 100 High better Based on data from 268 participants in 2 studies	Difference: **MD 0.74 higher** (CI 95% 14.66 lower - 16.15 higher)		**Low** Due to serious risk of bias, Due to serious imprecision	CBT may have little or no effect on mental health compared to usual care.
Mental Health (Beck Depression Inventory)	Scale: 0 - 63 Lower better Based on data from 43 participants in 1 study	Difference: **MD 0.43 lower** (CI 95% 2.82 lower - 1.96 higher)		**Low** Due to serious risk of bias, Due to serious imprecision	CBT may have little or no effect on mental health (depression) compared to usual care.
Cognitive Function (Delayed Recall 15 Words Test)	Scale: 0 - 15 High better Based on data from 180 participants in 1 study	Difference: **MD 0.75 higher** (CI 95% 0.19 lower - 1.68 higher)		**Low** Due to serious risk of bias, Due to serious imprecision	CBT may have little or no effect on cognitive function compared to usual care.
Signs and Symptoms of Insomnia (Insomnia Severity Index)	Scale: 0 - 28 Lower better Based on data from 168 participants in 3 studies	Difference: **MD 1.56 lower** (CI 95% 4.41 lower - 1.29 higher)		**Moderate** Due to serious risk of bias	CBT probably has little or no effect on signs and symptoms of insomnia compared to usual care.

Sleep Symptoms and Sleep Efficiency (Pittsburgh Sleep Quality Index)	Scale: 0 - 21 Lower better<br>Based on data from 152 participants in 2 studies	Difference: **MD 0.51 higher**<br>(CI 95% 5.46 lower - 6.48 higher)	**Very low**<br>Due to serious risk of bias, Due to serious imprecision, Due to serious inconsistency	We are uncertain of the effects of CBT on sleep symptoms and sleep efficiency compared to usual care.
Total Sleep Time (Hours)	Scale: High better<br>Based on data from 281 participants in 4 studies	Difference: **MD 0.51 lower**<br>(CI 95% 1.19 lower - 0.18 higher)	**Low**<br>Due to serious risk of bias, Due to serious imprecision	CBT may have little or no effect on total sleep time compared to usual care.
Sleep Onset Latency (Minutes)	Scale: Lower better<br>Based on data from 219 participants in 3 studies	Difference: **MD 5.46 higher**<br>(CI 95% 6.35 lower - 17.26 higher)	**Moderate**<br>Due to serious risk of bias	CBT probably has little or no effect on sleep onset latency compared to usual care.
Sleep Efficiency	Scale: 0 - 100 High better<br>Based on data from 119 participants in 2 studies	Difference: **MD 1.13 lower**<br>(CI 95% 7.79 lower - 5.52 higher)	**Moderate**<br>Due to serious risk of bias	CBT probably has little or no effect on sleep efficiency compared to usual care.
Drug Free Nights<br>7 nights	Scale: High better<br>Based on data from 102 participants in 1 study	Difference: **MD 1.8 higher**<br>(CI 95% 0.64 higher - 2.96 lower)	**Low**<br>Due to serious risk of bias, Due to serious imprecision	CBT may increase drug-free nights compared to usual care.

Supplement 21: Table of trial characteristics for trials that investigated medication review

Trial	Registration	Funding	Design	Country	Male (%)	Mean age	Mean duration of benzodiazepine use/sedative hypnotics (years)	Using benzodiazepines/ sedative hypnotics for insomnia (%)	Using benzodiazepines/ sedative hypnotics for anxiety (%)	Using benzodiazepines/ sedative hypnotics for conditions other than insomnia (%)	Interventions	Number of patients	Description
**Meredith, 2002**	NR	Government, Non-profit	Parallel RCT	United States of America	74.9	80.05	NR	NR	NR	NR	Medication review	160	Multicomponent intervention for home healthcare patients; Clinical pharmacists were trained and managed the intervention at each home care site; Clinical pharmacists prepared educational materials for nurses to explain potential medication problems; Pharmacist and patient's nurse jointly reviewed identified medication problems for each patient; Problems and solutions were passed on to the patient's physician using structured templates; Physicians provided instructions for changes in therapy and nurses administered changes; Clinical pharmacologists were available for consultations, when necessary
											usual care	157	Usual care
**Strauven, 2019 (COME-ON)**	ISRCTN66138978	Government	Cluster RCT	Belgium	29.49	87 (medication review), 88 (usual care)*	≥ 0.08	NR	NR	NR	medication review, education (physicians, nurses, pharmacists)	847	Multicomponent intervention targeting nursing home physicians, pharmacists, and nurses, and consisted of a blended training program, interdisciplinary meetings, and interdisciplinary case conferences (ICC). Blended training program involved e-learning and face-to-face workshops, including topics on pharmacotherapy for

											usual care	957	older people and medication review. Interdisciplinary meetings involved discussing the rational use of a single medication class at the nursing home level. ICCs consisted of structured and repeated interdisciplinary face-to-face medication reviews (every 4 months for 12-months) of residents and interventions to optimize medication use
**Sibille, 2021, 2023 (OPERAM)**	NCT02986425	Government	Cluster RCT	Belgium, Ireland, Netherlands, Switzerland	55.4 (mix of users and non-users)	78 (mix of users and non-users)*	NR	NR	NR	NR	medication review (physicians)	172	Patients received STRIP--an in-hospital digital structured pharmacotherapy optimization program, consisting of medication history; recording medication use and diagnoses; medication review using integrated STOPP/START criteria; and discussion with physician and patient to adapt recommendations
											usual care	206	Usual care

NR=Not reported
*Median

Supplement 22: Summary of findings for the comparison of medication review against usual care

Outcome	Study results and measurements	Absolute effect estimates		Certainty of the evidence (Quality of evidence)	Summary
		Usual Care	Medication Review		
Discontinuation	Odds ratio: 2.04 (CI 95% 1.31 - 3.17) Based on data from 736 participants in 2 studies	**130** per 1000	**234** per 1000	**Low** Due to serious risk of bias, Due to serious imprecision	Medication review may increase the proportion of patients who discontinue benzodiazepines/related hypnotics compared to usual care.
		Difference: **104 more per 1000** (CI 95% 34 more - 191 more)			

Supplement 23: Table of trial characteristics for trials that investigated mindfulness

Trial	Registration	Funding	Design	Country	Male (%)	Mean age	Mean duration of benzodiazepine use/sedative hypnotics (years)	Using benzodiazepines/ sedative hypnotics for insomnia (%)	Using benzodiazepines/ sedative hypnotics for anxiety (%)	Using benzodiazepines/ sedative hypnotics for conditions other than insomnia (%)	Interventions	Number of patients	Description
**Barros, 2021**	NCT02127411	Government, Non-profit	Parallel RCT	Brazil	0	54	4.5	100	NR	0	mindfulness	36	Motivational interviewing performed by a researcher that included feedback to patients on hypnotics and hazards, advice on all possible options, and helping patients realize they can take action; Eight weekly sessions of group mindfulness-based relapse prevention; Patients received a CD for guided home meditation
											motivational interviewing	34	Motivational interviewing performed by a researcher that included feedback to patients on hypnotics and hazards, advice on all possible options, and helping patients realize they can take action

NR=Not reported
*Median

Supplement 24: Summary of findings for the comparison of mindfulness against motivational interviewing

Outcome	Study results and measurements	Absolute effect estimates		Certainty of the evidence (Quality of evidence)	Summary
		Motivational Interviewing	Mindfulness		
Dropouts During Intervention	Odds ratio: 2.07 (CI 95% 0.47 - 9.02) Based on data from 70 participants in 1 study	**110** per 1000	**204** per 1000	**Very low** Due to serious risk of bias, Due to very serious imprecision	We are uncertain of the effects of mindfulness on dropouts during intervention compared to motivational interviewing.
		Difference: **94 more per 1000** (CI 95% 55 fewer - 417 more)			
Signs and Symptoms of Insomnia (Insomnia Severity Index)	Measured by: Scale: 0 - 28 Lower better Based on data from 52 participants in 1 study	Difference: **MD 4.82 lower** (CI 95% 7.87 lower - 1.77 lower)		**Low** Due to serious risk of bias, Due to serious imprecision	Mindfulness may have little or no effect on signs and symptoms of insomnia compared to motivational interviewing.

Supplement 25: Table of trial characteristics for trials that investigated pharmacist-led interventions

Trial	Registration	Funding	Design	Country	Male (%)	Mean age	Mean duration of benzodiazepine use/sedative hypnotics (years)	Using benzodiazepines/ sedative hypnotics for insomnia (%)	Using benzodiazepine s/sedative hypnotics for anxiety (%)	Using benzodiazepines/ sedative hypnotics for conditions other than insomnia (%)	Interventions	Number of patients	Description
**Roberts, 2001**	NR	Government	Cluster RCT	Australia	NR	NR	NR	NR	NR	NR	clinical pharmacy services in nursing homes	905	Introduced clinical pharmacy services in nursing homes over 12 months; 6 to 9 problem-based education sessions for nurses totalling 11 hours addressing geriatric pharmacology and problems in long term care; Pharmacist-led medication reviews of 500 residents addressing adverse drug effects, ceasing one or more drugs, adding drugs, better use of specific drug therapy, particularly psychoactive drugs, nondrug interventions, and adverse effect and drug response monitoring; Indirect physician involvement through existing relationships and focus groups and interviews facilitated by research nurse
											usual care	2325	Usual care
**van de Steeg-van Gompel, 2009**	NCT00268775	Industry, Institutional	Cluster RCT	Netherlands	30.18	64.88	≥0.25	NR	NR	NR	multicomponent intervention for pharmacists	11423	A multicomponent intervention in which pharmacists identified long term benzodiazepine users and worked with physicians to send discontinuation letters to patients asking them to contact their pharmacist or physician to discontinue; Pharmacists received an educational manual with an example discontinuation letter, instructions on managing the project, schedules for reducing benzodiazepines, information on long term effects of benzodiazepines and the effectiveness of letters for patients; Researchers arranged a six hour interactive

													educational meeting for the needs of the pharmacists addressing working and communicating with physicians; A coach called pharmacists to remind the pharmacists to get started on the intervention
											educational intervention for pharmacists	7975	Pharmacists identified long term benzodiazepine users and worked with physicians to send discontinuation letters to patients asking patients to contact their pharmacist or physician to discontinue; Educational manual with instructions on managing the project, schedules for reducing benzodiazepines, information on long term effects of benzodiazepines and the effectiveness of letters for patients
**Martin, 2018 (D-PRESCRIBE)**	NCT02053194	Government	Cluster RCT	Canada	34.15	74.7	10.53	NR	NR	NR	pharmacist-led education (patients, physicians)	248	Pharmacists were encouraged to distribute patient and physician education materials in-person or by mail; Patient educational materials included drug-specific brochure describing rationale why drug may be inappropriate, alternative treatment options, and a visual tapering protocol; Physician educational materials were developed based on input by pharmacists and physicians and included rationale for deprescribing, evidence about drug harms, credible sources of recommendations, safer alternatives, and personalized participant data (including patient names, drugs targeted, indication, and rationale behind the opinion)
											usual care	241	Usual care

NR=Not reported
*Median

Supplement 26: Summary of findings for the comparison of pharmacist-led educational intervention against usual care

Outcome	Study results and measurements	Absolute effect estimates		Certainty of the evidence (Quality of evidence)	Summary
		Usual Care	Pharmacist-led education (patients, physicians)		
Discontinuation	Relative risk: 4.78 (CI 95% 2.8 - 8.14) Based on data from 301 participants in 1 study	**130** per 1000	**621** per 1000	**Low** Due to serious risk of bias, Due to serious imprecision	Pharmacist-led educational intervention may increase the proportion of patients who discontinue benzodiazepines/sedative hypnotics compared to usual care.
		Difference: **491 more per 1000** (CI 95% 234 more - 928 more)			

Supplement 27: Summary of findings for the comparison of a multicomponent intervention involving pharmacists against an educational intervention for pharmacists

Outcome	Study results and measurements	Absolute effect estimates: Educational Intervention for Pharmacists	Absolute effect estimates: Multicomponent Intervention for Pharmacists	Certainty of the evidence (Quality of evidence)	Summary
Discontinuation	Odds ratio: 1.05 (CI 95% 0.36 - 3.13) Based on data from 19398 participants in 1 study	**100** per 1000	**104** per 1000	**Low** Due to serious risk of bias, Due to serious imprecision	Multicomponent intervention involving pharmacists may have little or no effect on the proportion of patients who discontinue benzodiazepines/sedative hypnotics compared to an education intervention for pharmacists.
		Difference: **4 more per 1000** (CI 95% 62 fewer - 158 more)			

Supplement 28: Summary of findings for the comparison of the introduction of clinical pharmacy services in nursing homes against usual care

**Outcome**	**Study results and measurements**	**Absolute effect estimates** Usual care	Clinical pharmacy services in nursing homes	**Certainty of the evidence** (Quality of evidence)	**Summary**
Number of Prescriptions (per 1000 patients per year)	Scale: Lower better Based on data from 3230 participants in 1 study	Difference: **MD 875 lower** (CI 95% 1634.8 lower - 115.2 lower)		**Moderate** Due to serious risk of bias	Clinical pharmacy services in nursing homes probably reduce the number of prescriptions of BSH compared to usual care.

Supplement 29: Table of trial characteristics for trials that investigated pharmacologic-assisted tapering

Trial	Registration	Funding	Design	Country	Male (%)	Mean age	Mean duration of benzodiazepine use/sedative hypnotics (years)	Using benzodiazepines/ sedative hypnotics for insomnia (%)	Using benzodiazepines/ sedative hypnotics for anxiety (%)	Using benzodiazepines/ sedative hypnotics for conditions other than insomnia (%)	Interventions	Number of patients	Description
**Tyrer, 1981**	NR	Government	Parallel RCT	United Kingdom	NR	NR	3.6	NR	NR	NR	propranolol, abrupt withdrawal	20	Abrupt withdrawal of benzodiazepine and replacement with 20 mg of propranolol three times a day for one week, increasing if necessary to 40 mg three times a day at week two
											abrupt withdrawal	20	Abrupt withdrawal of benzodiazepine
**Murphy, 1991**	NR	Industry	Parallel RCT	United Kingdom	23.53	44.93	7.67	NR	NR	NR	diazepam, taper	22	Patients switched to diazepam in roughly same dose as original benzodiazepine; Tapering (25% reduction of dosage at 2-week intervals, full suppression by week 10)
											lorazepam, taper	23	Patients switched to lorazepam in roughly same dose as original benzodiazepine; Tapering (25% reduction of dosage at 2-week intervals full suppression by week 10)
											bromazepam, taper	23	Patients switched to bromazepam in roughly same dose as original benzodiazepine; Tapering (25% reduction of dosage at 2-week intervals full suppression by week 10)
**Tyrer, 1996**	NR	Industry	Parallel RCT	United Kingdom	NR	NR	10*	NR	NR	NR	dothiepin, taper	41	Tapering (20% reduction of dosage at 2-week intervals, full suppression by week 8); 25 mg of dothiepin at week 0, 75 mg by week 2, and 150 mg by week 4
											taper	46	Tapering (20% reduction of dosage at 2-week intervals, full suppression by week 8)

**Cardinali, 2002**	NR	Industry, Government, Institutional	Parallel RCT	Argentina	20	70.5	NR	100	NR	NR	melatonin, taper	25	Tapering (50% reduction of dosage at 2-week intervals, full suppression by week 4); 3 mg melatonin daily for 6 weeks
											taper	23	Tapering (50% reduction of dosage at 2-week intervals, full suppression by week 4)
**Gerra, 2002**	NR	NR	Parallel RCT	NR	52	36.72	3 to 4	NR	NR	NR	flumazenil, oxazepam	20	Daily one-hour meetings with counsellor and psychiatrist using CBT; Flumazenil (1 mg twice per day); Oxazepam (7.5 mg once to twice per day) for the first 3 nights
											oxazepam	20	Daily one-hour meetings with counsellor and psychiatrist using CBT; Oxazepam (tapering over 8 days starting at 105 mg)
**Petrovic, 2002**	NR	NR	Parallel RCT	Belgium	32.5	81.5	≥0.25	75	25	0	lormetazepam, abrupt withdrawal	20	Benzodiazepines replaced with 1 mg lormetazepam administered at bedtime for 1 week; Abrupt withdrawal following first week
											abrupt withdrawal	20	Benzodiazepines replaced with placebo for 1 week; Abrupt withdrawal following first week
**Nakao, 2006**	NR	NR	Parallel RCT	Japan	33.33	58.5	6.03	NR	NR	NR	paroxetine, taper	22	Tapering (25% reduction of dosage at 2-week intervals); 10 to 20 mg paroxetine a day for 8 weeks
											taper	23	Tapering (25% reduction of dosage at 2-week intervals)
											usual care	21	Usual care
**Zee, 2008**	NCT00492232	Industry	Parallel RCT	United States of America	44	49.2	>0.25	100	0	0	ramelteon, taper	65	Ramelteon 8 mg, tablets, orally, once daily and current zolpidem therapy incrementally reduced by dose, frequency, or both for up to 10 weeks
											taper	70	Tapering
**Lähteenmäki,**	EudraCT20080	Industry,	Parallel RCT	Finland	33.7	66.5	9.9	100	0	0	melatonin,	46	Tapering (25% to 50%

**2013**	00679530	Government, Institutional, Non-profit	(melatonin, taper), 65 (taper)*	taper		reduction of dosage at 1 week intervals); 2 mg controlled release melatonin; Patients received sleep hygiene counselling and psychosocial support by physician, including discussion on regular sleep rhythm and the influence of aging, the bedroom, eating and alcohol, coffee and stimulants, exercise, breathing, and physical relation on sleep; Psychosocial support was continued by nurse including weekly visits and availability on phone
				taper	46	Tapering (25% to 50% reduction of dosage at 1-week intervals); Patients received sleep hygiene counselling and psychosocial support by physician, including discussion on regular sleep rhythm and the influence of aging, the bedroom, eating and alcohol, coffee and stimulants, exercise, breathing, and physical relation on sleep; Psychosocial support was continued by nurse including weekly visits and availability on phone

NR=Not reported
*Median

Supplement 30: Summary of findings for the comparison of tapering with melatonin against tapering alone

Outcome	Study results and measurements	Absolute effect estimates		Certainty of the evidence (Quality of evidence)	Summary
		Tapering alone	Tapering with melatonin		
Discontinuation	Odds ratio: 0.77 (CI 95% 0.3 - 1.96) Based on data from 83 participants in 1 study	**204** per 1000	**165** per 1000	**Very low** Due to serious risk of bias, Due to very serious imprecision	We are uncertain of the effects of tapering with melatonin on the proportion of patients who discontinue benzodiazepines/sedative hypnotics compared to tapering alone.
		Difference: **39 fewer per 1000** (CI 95% 133 fewer - 130 more)			
Dropouts During Intervention	Odds ratio: 1.0 (CI 95% 0.06 - 16.48) Based on data from 92 participants in 1 study	**137** per 1000	**137** per 1000	**Very low** Due to extremely serious imprecision	We are uncertain of the effects of tapering with melatonin on dropouts during intervention compared to tapering alone.
		Difference: **0 fewer per 1000** (CI 95% 128 fewer - 586 more)			
Sleep Onset Latency (Minutes)	Scale: Lower better Based on data from 45 participants in 1 study	Difference: **MD 0.71 lower** (CI 95% 1.43 lower - 0.01 higher)		**Low** Due to serious risk of bias, Due to serious imprecision	Tapering with melatonin may have little or no effect on sleep onset latency compared to tapering alone.

Supplement 31: Summary of findings for the comparison of tapering with paroxetine against tapering alone

Outcome	Study results and measurements	Absolute effect estimates: Tapering alone	Absolute effect estimates: Tapering with paroxetine	Certainty of the evidence (Quality of evidence)	Summary
Discontinuation	Odds ratio: 3.96 (CI 95% 1.01 - 15.52) Based on data from 45 participants in 1 study	**204** per 1000	**504** per 1000	**Very low** Due to serious risk of bias, Due to very serious imprecision	We are uncertain of the effects of tapering with paroxetine on the proportion of patients who discontinue benzodiazepine/related hypnotics compared to tapering alone.
		Difference: **300 more per 1000** (CI 95% 2 more - 595 more)			
Mental Health (Hamilton Rating Scale for Anxiety)	Measured by: Scale: 0 - 56 Lower better Based on data from 45 participants in 1 study	Difference: **MD 5.20 lower** (CI 95% 9.98 lower - 0.42 lower)		**Low** Due to serious risk of bias, Due to serious imprecision	Tapering with paroxetine may improve mental health (anxiety) compared to tapering alone.
Mental Health (Hamilton Rating Scale for Depression)	Measured by: Scale: 0 - 53 Lower better Based on data from 45 participants in 1 study	Difference: **MD 3.20 lower** (CI 95% 5.74 lower - 0.66 lower)		**Low** Due to serious risk of bias, Due to serious imprecision	Tapering with paroxetine may improve mental health (depression) compared to tapering alone.

Supplement 32: Summary of findings for the comparison of tapering with ramelteon against tapering alone

Outcome	Study results and measurements	Absolute effect estimates		Certainty of the evidence (Quality of evidence)	Summary
		Tapering alone	Tapering with ramelteon		
Discontinuation	Odds ratio: 0.84 (CI 95% 0.36 - 1.95) Based on data from 101 participants in 1 study	**204** per 1000	**177** per 1000	**Very low** Due to serious risk of bias, Due to very serious imprecision	We are uncertain of the effects of tapering with ramelteon on the proportion of patients who discontinue benzodiazepine/related hypnotics compared to tapering alone.
		Difference: **27 fewer per 1000** (CI 95% 120 fewer - 129 more)			
Dropouts During Intervention	Odds ratio: 0.69 (CI 95% 0.33 - 1.43) Based on data from 135 participants in 1 study	**137** per 1000	**99** per 1000	**High**	Tapering with ramelteon has little or no effect on dropouts during intervention compared to tapering alone.
		Difference: **38 fewer per 1000** (CI 95% 87 fewer - 48 more)			

Supplement 33: Summary of findings for the comparison of tapering with dothiepin against tapering alone

**Outcome**	**Study results and measurements**	**Absolute effect estimates**		**Certainty of the evidence** (Quality of evidence)	**Summary**
		Tapering alone	Tapering with dothiepin		
Dropouts During Intervention	Odds ratio: 0.78 (CI 95% 0.34 - 1.82) Based on data from 87 participants in 1 study	**204** per 1000	**167** per 1000	**Low** Due to very serious imprecision	Tapering with dothiepin may have little or no effect on dropouts during intervention compared to tapering alone.
		Difference: **37 fewer per 1000** (CI 95% 124 fewer - 114 more)			

Supplement 34: Summary of findings for the comparison of tapering with flumazenil and oxazepam against oxazepam alone

Outcome	Study results and measurements	Absolute effect estimates		Certainty of the evidence (Quality of evidence)	Summary
		Oxazepam	Flumazenil, Oxazepam		
Discontinuation	Odds ratio: 3.5 (CI 95% 0.94 - 12.97) Based on data from 40 participants in 1 study	**300** per 1000	**600** per 1000	**Very low** Due to serious risk of bias, Due to very serious imprecision	We are very uncertain of the effects of flumazenil and oxazepam on the discontinuation of benzodiazepines and closely related hypnotics compared to oxazepam alone.
		Difference: **300 more per 1000** (CI 95% 13 fewer - 548 more)			

Supplement 35: Summary of findings for the comparison of abrupt withdrawal with propranolol against abrupt withdrawal alone

Outcome	Study results and measurements	Absolute effect estimates		Certainty of the evidence (Quality of evidence)	Summary
		Abrupt withdrawal alone	Abrupt withdrawal with propranolol		
Dropouts During Intervention	Odds ratio: 2.27 (CI 95% 0.67 - 8.33) Based on data from 40 participants in 1 study	**350** per 1000	**550** per 1000	**Very low** Due to extremely serious imprecision	We are uncertain of the effects of abrupt withdrawal with propranolol on dropouts during intervention compared to abrupt withdrawal alone.
		Difference: **200 more per 1000** (CI 95% 85 fewer - 468 more)			

Supplement 36: Summary of findings for the comparison of switching to lormetazepam before abrupt withdrawal against abrupt withdrawal alone

Outcome	Study results and measurements	Absolute effect estimates		Certainty of the evidence (Quality of evidence)	Summary
		Abrupt Withdrawal	Lormetazepam		
Discontinuation	Odds ratio: 2.66 (CI 95% 0.65 - 11.11) Based on data from 40 participants in 1 study	**200** per 1000	**399** per 1000	**Very low** Due to extremely serious imprecision	We are uncertain of the effects of abrupt withdrawal with lormetazepam on the proportion of patients who discontinue of benzodiazepines/sedative hypnotics compared to abrupt withdrawal alone.
		Difference: **199 more per 1000** (CI 95% 60 fewer - 535 more)			
Dropouts During Intervention	Odds ratio: 0.25 (CI 95% 0.06 - 1.02) Based on data from 40 participants in 1 study	**500** per 1000	**200** per 1000	**Very low** Due to extremely serious imprecision	We are uncertain of the effects of withdrawal with lormetazepam on dropouts during intervention compared to abrupt withdrawal alone.
		Difference: **300 fewer per 1000** (CI 95% 443 fewer - 5 more)			
Sleep Symptoms and Sleep Efficiency (Pittsburgh Sleep Quality Index)	Measured by: Scale: 0 - 21 Lower better Based on data from 26 participants in 1 study	Difference: **MD 1.08 lower** (CI 95% 2.16 lower - 0.00 higher)		**Low** Due to serious risk of bias, Due to serious imprecision	Abrupt withdrawal with lormetazepam may have little or no effect on sleep symptoms and sleep efficiency compared to abrupt withdrawal alone.

Supplement 37: Summary of findings for the comparison of switching to bromazepam before tapering against switching to diazepam before tapering

Outcome	Study results and measurements	Absolute effect estimates<br>Diazepam	 Bromazepam	Certainty of the evidence (Quality of evidence)	Summary
Discontinuation	Odds ratio: 0.7 (CI 95% 0.2 - 2.51) Based on data from 45 participants in 1 study	**727** per 1000	**651** per 1000	**Very low** Due to extremely serious imprecision	We are uncertain of the effects of switching to bromazepam before tapering on the proportion of patients who discontinue benzodiazepines/sedative hypnotics compared to switching to diazepam.
		Difference: **76 fewer per 1000** (CI 95% 379 fewer - 143 more)			
Dropouts During Intervention	Odds ratio: 1.17 (CI 95% 0.32 - 4.25) Based on data from 45 participants in 1 study	**273** per 1000	**305** per 1000	**Very low** Due to extremely serious imprecision	We are uncertain of the effects of switching to bromazepam before tapering on dropouts during the intervention compared to switching to diazepam.
		Difference: **32 more per 1000** (CI 95% 166 fewer - 342 more)			

Supplement 38: Summary of findings for the comparison of switching to bromazepam before tapering against switching to lorazepam before tapering

Outcome	Study results and measurements	Absolute effect estimates<br>Lorazepam	 Bromazepam	Certainty of the evidence (Quality of evidence)	Summary
Discontinuation	Odds ratio: 1.44 (CI 95% 0.44 - 4.74) Based on data from 46 participants in 1 study	**565** per 1000	**652** per 1000	**Very low** Due to extremely serious imprecision	We are uncertain of the effects of switching to bromazepam before tapering on the proportion of patients who discontinue benzodiazepines/sedative hypnotics compared to switching to lorazepam.
		Difference: **87 more per 1000** (CI 95% 201 fewer - 295 more)			
Dropouts During Intervention	Odds ratio: 0.57 (CI 95% 0.17 - 1.91) Based on data from 46 participants in 1 study	**435** per 1000	**305** per 1000	**Very low** Due to extremely serious imprecision	We are uncertain of the effects of switching to bromazepam before tapering on dropouts during intervention compared to switching to lorazepam.
		Difference: **130 fewer per 1000** (CI 95% 319 fewer - 160 more)			

Supplement 39: Summary of findings for the comparison of switching to diazepam before tapering against switching to lorazepam before tapering

Outcome	Study results and measurements	Absolute effect estimates Lorazepam	Absolute effect estimates Diazepam	Certainty of the evidence (Quality of evidence)	Summary
Discontinuation	Odds ratio: 2.05 (CI 95% 0.59 - 7.15) Based on data from 45 participants in 1 study	**565** per 1000	**727** per 1000	**Very low** Due to extremely serious imprecision	We are uncertain of the effects of switching to diazepam before tapering on the proportion of patients who discontinue benzodiazepines/sedative hypnotics compared to switching to lorazepam.
		Difference: **162 more per 1000** (CI 95% 131 fewer - 338 more)			
Dropouts During Intervention	Odds ratio: 0.49 (CI 95% 0.14 - 1.7) Based on data from 45 participants in 1 study	**435** per 1000	**274** per 1000	**Very low** Due to extremely serious imprecision	We are uncertain of the effects of switching to diazepam before tapering on dropouts during intervention compared to switching to lorazepam.
		Difference: **161 fewer per 1000** (CI 95% 338 fewer - 132 more)			

Supplement 40: Summary of findings for the comparison of multicomponent interventions against single component interventions

Outcome	Study results and measurements	Absolute effect estimates: Single Component	Absolute effect estimates: Multicomponent	Certainty of the evidence (Quality of evidence)	Summary
Discontinuation	Odds ratio: 2.67 (CI 95% 0.92 - 7.78) Based on data from 19931 participants in 9 studies	**400** per 1000	**640** per 1000	**Low** Due to serious risk of bias, Due to serious imprecision	Multicomponent interventions may increase the proportion of patients who discontinue benzodiazepines/sedative hypnotics compared to single component interventions.
		Difference: **240 more per 1000** (CI 95% 20 fewer - 438 more)			
Dropouts During Intervention	Odds ratio: 1.26 (CI 95% 0.88 - 1.99) Based on data from 672 participants in 8 studies	**110** per 1000	**135** per 1000	**Low** Due to serious risk of bias, Due to serious imprecision	Multicomponent interventions may have little or no effect on dropouts during intervention compared to single component interventions.
		Difference: **25 more per 1000** (CI 95% 12 fewer - 87 more)			
